# Runx-mediated regulation of CCL5 via antagonizing two enhancers influences immune cell function and anti-tumor immunity

**DOI:** 10.1038/s41467-020-15375-w

**Published:** 2020-03-26

**Authors:** Wooseok Seo, Kanako Shimizu, Satoshi Kojo, Arinze Okeke, Terumi Kohwi-Shigematsu, Shin-ichiro Fujii, Ichiro Taniuchi

**Affiliations:** 1 Laboratory for Transcriptional Regulation, RIKEN Center for Integrative Medical Sciences, 1-7-22, Suehiro-cho, Tsurumi-ku, Yokohama, 230-0045 Japan; 2 Laboratory for Immunotherapy, RIKEN Center for Integrative Medical Sciences, 1-7-22, Suehiro-cho, Tsurumi-ku, Yokohama, 230-0045 Japan; 3 Department of Orofacial Sciences, University of California, San Francisco, CA 94143 USA; 4 Present Address: Department of Immunology, Nagoya University Graduate School of Medicine, Showa-ku Tsurumai-Cho 65, Nagoya, 466-8550 Japan

**Keywords:** Tumour immunology, Chemokines, Gene regulation in immune cells, Lymphocytes

## Abstract

CCL5 is a unique chemokine with distinct stage and cell-type specificities for regulating inflammation, but how these specificities are achieved and how CCL5 modulates immune responses is not well understood. Here we identify two stage-specific enhancers: the proximal enhancer mediates the constitutive CCL5 expression during the steady state, while the distal enhancer located 1.35 Mb from the promoter induces CCL5 expression in activated cells. Both enhancers are antagonized by RUNX/CBFβ complexes, and SATB1 further mediates the long-distance interaction of the distal enhancer with the promoter. Deletion of the proximal enhancer decreases CCL5 expression and augments the cytotoxic activity of tissue-resident T and NK cells, which coincides with reduced melanoma metastasis in mouse models. By contrast, increased CCL5 expression resulting from RUNX3 mutation is associated with more tumor metastasis in the lung. Collectively, our results suggest that RUNX3-mediated CCL5 repression is critical for modulating anti-tumor immunity.

## Abstract (web summary)

CCL5 is an important chemokine for modulation of inflammatory responses, but how CCL5 expression is regulated is still unclear. Here the authors show that the CCL5 locus contains two enhancers, with the proximal enhancer being responsible for homeostatic expression and the distal enhancer enforcing inducibility, while both enhancers are modulated by RUNX3.

## Introduction

Typical CC chemokines, such as CCL3 (MIP-1α) and CCL4 (MIP-1β), are rapidly expressed within a couple of hours after stimulation to attract myeloid^1^ and effector T cells to inflamed sites^2^. In contrast, CCL5, also known as RANTES (Regulated on Activation, Normal T cell Expressed and Secreted), is induced during the later stage of inflammation^3,4^ specifically to recruit activated effector T cells and freshly generated memory T cells^5^, which require several days to be produced. In addition to this induced expression during activation, CCL5 is also constitutively expressed in noninflamed conditions by memory T cells^6,7^ and local natural killer (NK) cells^8^. Recent studies using neutralizing anti-CCL5 antibodies have suggested that the constitutive expression of CCL5 plays a regulatory role in maintaining tissue-resident memory (Trm) T cells in the human vaginal tract^9^ and mouse skin^10^, suggesting that constitutive CCL5 expression in noninflamed conditions, hereafter referred to as homeostatic CCL5 expression, might be important for homeostasis of tissue-resident lymphocytes.

In addition to its roles in the regulation of inflammatory diseases and the maintenance of local immune cells, CCL5 expressed by cancer cells plays diverse roles in shaping cancer microenvironments toward their own survival. For example, several types of cancer cells express CCL5 to specifically recruit anti-inflammatory immune cells, such as regulatory T cells (Tregs)^11–13^, which can suppress antitumor immunity or to directly facilitate their own metastasis^14^. Other studies reported that some cancer cells could repress their own CCL5 expression to hinder migration of anticancer immune cells toward cancer sites^15,16^. Thus, cancer-derived CCL5 are generally utilized to evade host’s antitumor responses, representing a procancer role of cancer-secreted CCL5.

On the other hand, host-derived CCL5 has rather controversial (and contradicting) functions during tumorigenesis and metastasis. CCL5-deficient NK and helper T cells (Th) were shown to be less efficient in recruiting dendritic cells (DCs) and cytotoxic T lymphocytes to cancer sites, resulting in increased cancer development^17–19^. Therefore, host CCL5 can obviously work as an anticancer molecule to mount relevant immune responses against some cancer cells. On the contrary, recent studies using CCL5 knockout mice showed that the total absence of host CCL5 exhibits increased antitumor immunity in breast cancer models, proposing a unique procancer role of host CCL5^20,21^. Furthermore, a recent clinical trial for a CCR5 inhibitor treatment to patients suffering from liver metastasis of colorectal cancer showed its effect on reducing tumor mass with better prognosis, suggesting CCL5–CCR5 axis might also provide a procancer activity^22^. Therefore, it might be safe to say that host CCL5 plays important roles in controlling cancer development but might create either pro- or anticancer environments according to the given situation, such as the type of cells that produce CCL5 and the type of cancers.

Despite the importance of CCL5 in inflammation, Trm T-cell homeostasis and tumor immunity, little is known about how the expression of the *Ccl5* gene is regulated. There might be cases in which the inactivation of all CCL5 by neutralizing anti-CCL5 antibodies or CCL5 knockout are not adequate to examine a particular function of CCL5 due to its unique biphasic expression with the clear stage specificity.

Here, we identify two transcriptional enhancers which confer the stage specificity (homeostatic and inducible) on CCL5. We further show that both enhancers are negatively regulated by RUNX/CBFβ transcription factor complexes. By generating the knockout mice for each enhancer, we are able to dissect the specific function of CCL5 at specific stages. Interestingly, the homeostatic CCL5 expression from the host’s immune cells has significant impacts on priming functional states of the immune cells at nonimmune tissues, such as lungs, resulting in altered tumor immunity against metastatic cancer. Thus, our study supports a procancer role of host CCL5 and reveals that CCL5 levels in nonimmune tissues, such as cancer microenvironments, could be important to modulate functional states of immune cells at local tissues.

## Results

### Repression of *Ccl5* expression by RUNX/CBFβ complexes

**Fig. 1:**
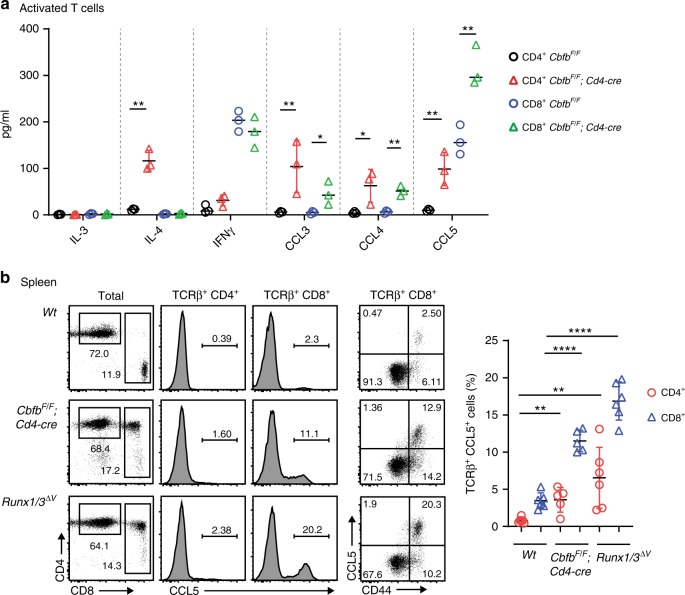
*Ccl5* expression from T cells is repressed by RUNX/CBFβ complexes. **a** Expression profiles assessed by ELISA of CCL3, CCL4, and CCL5 and the selected cytokines IL-3, IL-4, and IFNγ in supernatants of in vitro-stimulated CD4^+^ and CD8^+^ T cells at 5 days after stimulation. A summary of three independent measurements on three mice (with their genotypes indicated) are shown. Error bars indicate Mean ± SD and each dot represents a mouse examined over at least two independent experiments. Statistical significance is measured via unpaired two-tailed Student’s *t* tests and is presented as follows: *p < 0.05, **p < 0.01. **b** Flow cytometry analysis of total splenocytes and splenic TCRβ^+^ T cells in mice with their genotypes indicated. One representative profile of the CCL5 expression is shown in the histograms and dot plots. Numbers in the dot plots indicate the percentage of cells in each quadrant. Error bars indicate Mean ± SD and each dot represents a mouse examined over at least two independent experiments. Statistical significance is measured via unpaired two-tailed Student’s *t* tests and is presented as follows: **p < 0.01, ****p < 0.0001. Source data are provided as a Source Data file.

RUNX transcription factor family proteins hetero-dimerizing with CBFβ, an essential partner protein, play important roles in many developmental processes, such as hematopoiesis, and are involved in the pathogenesis of several inflammatory diseases, such as colitis^23^ and lung inflammation^24,25^. One of the causal mechanisms for these inflammatory phenotypes is higher IL-4 expression in activated T cells in the absence of RUNX/CBFβ^26^. Given the milder lung pathologies observed in IL-4 transgenic mice^27^, we examined whether inflammatory cytokines/chemokines, other than IL-4, are highly produced by CBFβ-deficient activated T cells. Of the 22 cytokines screened, CC chemokines, such as CCL3, CCL4, and CCL5, were secreted at higher levels from CBFβ-deficient cells than control cells, in addition to IL-4 and IL-5 (Supplementary Fig. 1a). An enzyme-linked immunosorbent assay (ELISA) using supernatants of activated T cells at 5 days after stimulation confirmed higher CCL5 secretion from activated CD8^+^ cytotoxic T cells (Tc) and CD4^+^ Th upon the loss of CBFβ (Fig. 1a), although the CCL5 expression is known to be induced mainly by activated Tc cells. This finding indicates that RUNX/CBFβ not only regulates the amounts but also the cell-type specificities of the CCL5 expression. The loss of CBFβ did not make any difference to CCL3 or CCL4 levels at day 2 after activation (Supplementary Fig. 1b). However, contrary to that in wild-type cells, the expression of CCL3 and CCL4 continued in Th cells in the absence of CBFβ, and was still detected even 7 days after activation (Supplementary Fig. 1b), indicating a role for RUNX/CBFβ in suppressing *Ccl3* and *Ccl4* expression at the later phase of T-cell activation.

CCL5 is a unique inflammatory chemokine with regard to its homeostatic expression in memory T and NK cells during steady states^6,7^. Indeed, we observed the CCL5 expression in T cells, specifically CD44^+^ memory T cells, as well as TCRβ^−^ non-T cells, in wild-type mice by flow cytometry (Supplementary Fig. 1c). Because RUNX/CBFβ complexes are crucial for *Cd4* gene silencing in CD8^+^ lineage T cells^28^ by recruiting transducin-like enhancer (TLE) of split corepressor family proteins through the C-terminal VWRPY penta-peptide motif in RUNX proteins^29^, CD8^+^ T cells emerge as CD4^+^CD8^+^ T cells in *Cbfb*^*F/F*^;*Cd4-Cre* mice and *Runx1/3*^*ΔV/ΔV*^ mice lacking the VWRPY-motif in both RUNX1 and RUNX3 proteins^30^. In such CD4^+^CD8^+^ T cells, the percentage of CCL5^+^ cells in the CD44^+^ population was over fivefold higher than in control cells (Fig. 1b). In addition, the ectopic CCL5 expression was induced in CD4^+^CD8^−^ T cells of those *Runx* mutant mice (Fig. 1b). *Runx1/3*^*ΔV/ΔV*^ mice also exhibited elevated CC chemokine secretion after in vitro stimulation (Supplementary Fig. 2a) as was observed in the activated T cells of *Cbfb*^*F/F*^;*Cd4-Cre* mice (Supplementary Fig. 1b). The greater secretion of CC chemokines from CD4^+^ T cells in *Runx3*^*ΔV/ΔV*^ mice than those of *Runx1*^*ΔV/ΔV*^ mice, albeit at lower levels than those from *Runx1/3*^*ΔV/ΔV*^ cells, indicates that RUNX3 plays a dominant role in suppressing CC chemokines, as well as the functional redundancy between RUNX1 and RUNX3 (Supplementary Fig. 2b). These observations demonstrate that RUNX/CBFβ complexes play a crucial role in regulating stage- and cell-type specific expression of the CC chemokine genes, *Ccl3/4/5*.

### A proximal enhancer regulates homeostatic *Ccl5* expression

**Fig. 2:**
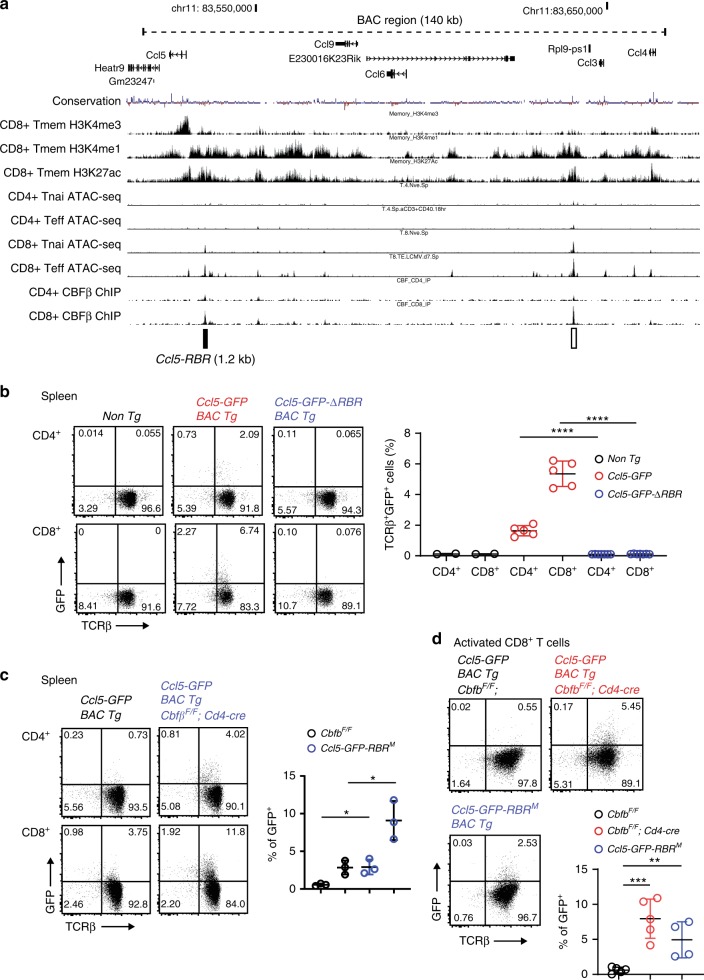
*Ccl5* expression is regulated by two stage-specific enhancers. **a** CBFβ ChIP-seq tracks in splenic total CD4^+^ and CD8^+^ T cells at one of the two CC chemokine clusters in which *Ccl5* is located on chromosome 11. ATAC-seq tracks in naïve (Tnai) and effector (Teff) T cells and histone mark ChIP-seq tracks in memory CD8^+^ (Tmem) T cells obtained from public database are also indicated. Two significant CBFβ ChIP-seq peaks are indicated by rectangles, one of which (near *Ccl5*) is denoted as *Ccl5-RBR*. *Ccl5-RBR* has a 0.2 kb core enhancer region denoted as *Ccl5-PE* that harbors two Runx recognition sequences (RRSs). The genome region included in the BAC transgene construction is indicated by a dotted line. CBFβ ChIP-seq data are representative of two independent experiments. **b** Representative dot plots showing GFP expression in splenocytes of F0 transgenic founders. A *Ccl5-GFP* BAC transgene was constructed by replacing *Ccl5* with *eGFP*. In the *Ccl5-GFP-ΔRBR* BAC transgene, a 1.2 kb RUNX binding region (RBR) near *Ccl5* (*Ccl5-RBR*) was removed from the *Ccl5-GFP* BAC transgene. The graph on the right shows a summary of frequencies of GFP^+^ cells in splenic T cells of each F0 transgenic founder. Error bars indicate Mean ± SD and each dot represents a mouse examined over at least two independent experiments. Statistical significance is measured via unpaired two-tailed Student’s *t* tests and is presented as follows: ****p < 0.0001. **c** Representative dot plots show representative GFP expression in spleen T cells of the *Ccl5-GFP* BAC transgenic founders in the presence or absence of RRSs. Numbers in the dot plots indicate the percentage of cells in each quadrant. Error bars indicate Mean ± SD and each dot represents a mouse examined over at least two independent experiments. Statistical significance is measured via unpaired two-tailed Student’s *t* tests and is presented as follows: *p < 0.05. **d** Representative dot plots showing GFP expression in activated CD8^+^ T cells. The left and right plots are of the *Ccl5-GFP* BAC transgenic founder in the presence and absence of CBFβ, respectively. The bottom plot shows the GFP expression in one representative F0 founder line of the *Ccl5-GFP*-*RBR*^*M*^ BAC transgene, in which two RUNX recognition sequences (RRSs) within the *Ccl5-RBR* are mutated. The graph shows a summary of the frequencies of GFP^+^ cells in activated CD8^+^ T cells. Error bars indicate Mean ± SD and each dot represents a mouse examined over at least two independent experiments. Statistical significance is measured via unpaired two-tailed Student’s *t* tests and is presented as follows: **p < 0.01, ***p < 0.001. Source data are provided as a Source Data file.

In order to understand the molecular mechanisms by which RUNX/CBFβ complexes suppress CC chemokine genes, we examined whether and where RUNX/CBFβ complexes associate around the *Ccl3/4/5* cluster on chromosome 11 by performing chromatin immune precipitation combined with deep-sequencing (ChIP-seq) using an anti-CBFβ antibody in splenic CD4^+^ and CD8^+^ T cells. ChIP-seq identified two genomic regions bound by RUNX/CBFβ, one at 5 kb upstream from the *Ccl5* transcription start site (TSS), which is referred to as *Ccl5-RBR* (RUNX bound region), and the other at 7 kb downstream of the 3′UTR of the *Ccl3* gene (Fig. 2a). Histone modifications, H3K4me1 and H3K27Ac, which are well-known markers for transcriptional enhancers, had accumulated in these regions (Fig. 2a), indicating that both regions have the potential to function as transcriptional enhancers. Consistent with the expression levels of CCL5, open chromatin structures of these regions analyzed by ATAC-seq are more pronounced in CD8^+^ T cells than CD4^+^ T cells (Fig. 2a). Interestingly, *Ccl5-RBR* is very well conserved between species (Supplementary Fig. 3c) and RUNX recognition sequences (RRSs; 5′-PuACCPuCA-3′) are clearly identified in *Ccl5-RBR* (Supplementary Fig. 3a, b). On the other hand, typical RRSs are not present in the RUNX/CBFβ ChIP-seq peak near *Ccl3* locus, suggesting that this peak could be resulted from indirect association of RUNX/CBFβ. As a note, the promoter region (±200 bp of TSS) of *Ccl5* also does not contain RRSs and does not have the peak signal in RUNX/CBFβ ChIP-seq.

To characterize the *Ccl5-RBR* region in more detail, we constructed a BAC transgene (Tg) that encompasses a 140 kb fragment, including the entire *Ccl3/4/5/6/9* gene cluster (Fig. 2a and Supplementary Fig. 3d). The BAC Tg constructs have an *eGFP* replacement with the *Ccl5* coding region in the presence (*Ccl5-GFP* BAC) or absence (*Ccl5-GFP*-*ΔRBR* BAC) of a 1.2 kb *Ccl5-RBR* (Supplementary Fig. 3d). In four out of five F0 founders that were generated with the control *Ccl5-GFP* BAC construct, we detected reporter *GFP* expression in non-T cells and T cells (Fig. 2b) at a comparable frequency as that of endogenous *Ccl5* expression as assessed by an anti-CCL5 antibody (Fig. 1b). In sharp contrast, *GFP* expression was not detected, or was very low, in all seven F0 founders that were generated using the *Ccl5-GFP-ΔRBR* BAC construct (Fig. 2b). However, we noticed that GFP expression in the *Ccl5-GFP* is lost after in vitro activation (Fig. 2d). These observations not only indicate that *Ccl5-RBR* is probably the main enhancer that controls homeostatic *Ccl5* expression in T cells during the steady state, but also that other genomic region(s) outside the region contained within the BAC construct must be involved in the induction of *Ccl5* for activated CD8^+^ T cells.

To ascertain whether and how RUNX complexes regulate *Ccl5-RBR* function, we monitored GFP expression in one representative *Ccl5-GFP* BAC Tg line in the absence of CBFβ expression by crossing with *Cbfb*^*F/F*^;*Cd4-Cre* mice. While CD4^+^ T cells of BAC tg lines does not show appreciable amounts of GFP after activation, we observed an increase in GFP-expressing cells without CBFβ expression by *Cd4-cre* (Fig. 2c), which is consistent with the higher endogenous *Ccl5* expression observed in the *Cbfb*^*F/F*^;*Cd4-Cre* mice (Fig. 1b). Interestingly, GFP expression in *Ccl5-GFP* BAC Tg was induced also by activated CD8^+^ T cells upon the loss of CBFβ (Fig. 2d). Moreover, when two RRSs in *Ccl5* proximal enhancer (*Ccl5-PE*) in the BAC Tg construct were mutated to inhibit RUNX/CBFβ binding (*Ccl5-GFP-RBR*^*M*^ BAC Tg) (Supplementary Fig. 3d), GFP expression was induced by activated CD8^+^ T cells (Fig. 2d). These observations clearly indicate that the enhancer activity as well as its stage specificity of *Ccl5-RBR* is suppressed by the direct binding of RUNX/CBFβ.

**Fig. 3:**
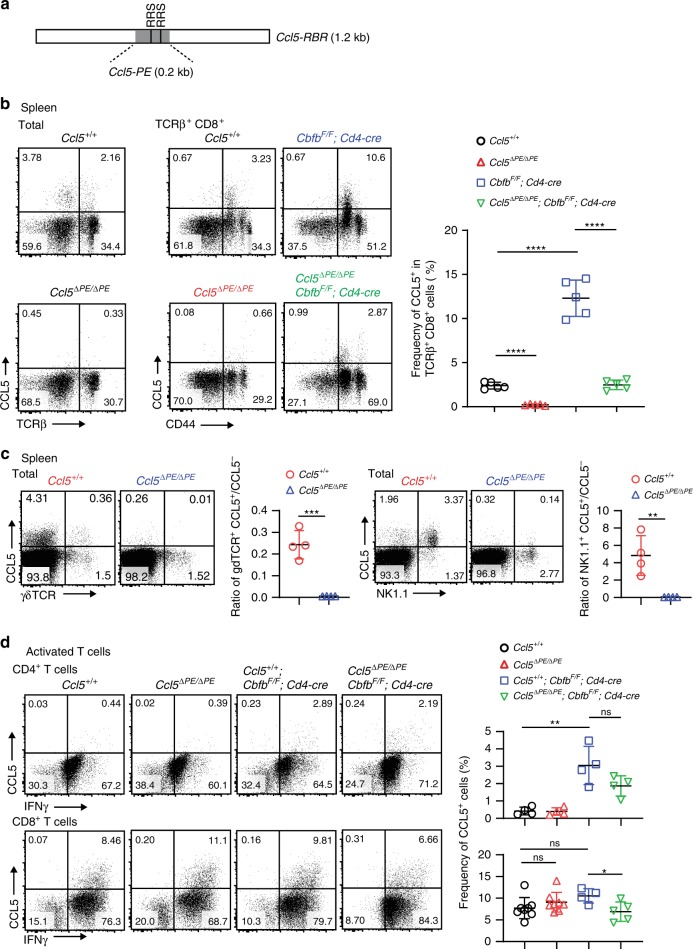
*Ccl5* proximal enhancer regulates homeostatic CCL5 expression. **a** A schematic representation of 0.2 kb *Ccl5-PE* in relation to 1.2 kb *Ccl5-RBR* (RUNX binding region). Two RRSs (RUNX recognition sites) within *Ccl5-PE* are shown as vertical lines. **b** Representative dot plots showing CCL5 expression in total and TCRβ^+^ splenocytes of mice with the indicated genotypes. In the *Ccl5*^*ΔPE*^ allele, a 185 bp core region in the 1.2 kb *Ccl5-RBR* region was deleted. The graph shows a summary of the frequencies of CCL5^+^ cells in splenic TCRβ^+^ T cells. Error bars indicate Mean ± SD and each dot represents a mouse examined over at least two independent experiments. Statistical significance is measured via unpaired two-tailed Student’s *t* tests and is presented as follows: ****p < 0.0001. **c** Representative dot plots of the CCL5 expression in γδ T and NK cells are shown with a summary graph that depicts the ratio of CCL5^+^ to CCL5^−^ in each cell type. Error bars indicate Mean ± SD and each dot represents a mouse examined over at least two independent experiments. Statistical significance is measured via unpaired two-tailed Student’s *t* tests and is presented as follows: **p < 0.01, ***p < 0.001. **d** Representative dot plots showing CCL5 expression in activated CD4^+^ T cells and activated CD8^+^ T cells at 5 days after in vitro stimulation. The graph shows a summary of the frequencies of CCL5^+^ cells in those cells. Numbers in the dot plots indicate the percentage of cells in each quadrant. Error bars indicate Mean ± SD and each dot represents a mouse examined over at least two independent experiments. Statistical significance is measured via unpaired two-tailed Student’s *t* tests and is presented as follows: *p < 0.05, **p < 0.01, ns nonsignificant. Source data are provided as a Source Data file.

Our results highlighted that an additional regulatory region(s) is necessary to induce the *Ccl5* expression from activated CD8^+^ T cells (Fig. 2d), in addition to the requirement of *Ccl5-RBR* during the steady state. Since this unknown enhancer element associated with activated status must be located outside of the 140 kb region inserted in the *Ccl5-GFP* BAC Tg construct, *Ccl5-RBR* is hereafter referred to as the *Ccl5* proximal enhancer (*Ccl5-PE)*, and the putative additional region(s) responsible for *Ccl5* induction in activated cells as the *Ccl5* distal enhancer (*Ccl5-DE*). In order to examine the physiological role that *Ccl5-PE* plays in regulating *Ccl5* expression, we generated a mutant mouse line in which the central 185 bp conserved element containing two RRSs is removed from the murine *Ccl5* locus by Crispr/Cas9-mediated genome editing (Fig. 3a). The homeostatic CCL5 expression in both T cells and non-T cells was lost upon removal of *Ccl5-PE* (*Ccl5*^*ΔPE/ΔPE*^ mice) (Fig. 3b), confirming that *Ccl5-PE* is the main component responsible for the enhancer activity of *Ccl5-RBR*. Also, in non-αβT cell population, we confirmed that both NK and γδT cells express homeostatic CCL5 and that the CCL5 expression in these cells was lost due to the lack of *Ccl5-PE* (Fig. 3c). NKT cells did not express CCL5 during the steady state (Supplementary Fig. 3e). In sharp contrast, the *Ccl5* expression in in vitro-activated CD8^+^ T cells was similar between *Ccl5*^*+/+*^ and *Ccl5*^*ΔPE/ΔPE*^ mice (Fig. 3d). This highlights an essential requirement for the presence of *Ccl5-DE* in activated cells, which was suggested by the loss of GFP signals from T cells of *Ccl5-GFP* BAC Tg after activation (Fig. 2d). In *Ccl5*^*ΔPE/ΔPE*^*; Cbfβ*^*F/F*^*; Cd4-cre* mice, the percentage of CCL5-expressing T cells was restored to a similar level as that in control *Ccl5*^*+/+*^ cells (Fig. 3b). Although CBFβ-deficiency did not have a significant impact on CCL5 induction from the *Ccl5*^*ΔPE*^ allele in activated CD8^+^ T cells (Tc condition), the CCL5 expression in activated CD4^+^ T cells (Th0 condition) was still increased by the loss of CBFβ (Fig. 3d). Therefore, even if *Ccl5* expression is controlled independently of *Ccl5-PE*, the loss of RUNX/CBFβ causes aberrant *Ccl5* expression in ex vivo CD8^+^ T cells and activated CD4^+^ T cells. These observations suggest that both the stage- and cell-type specificities of putative *Ccl5-DE* activity are also restrained by RUNX/CBFβ complexes.

### A distal enhancer regulates inducible *Ccl5* expression

**Fig. 4:**
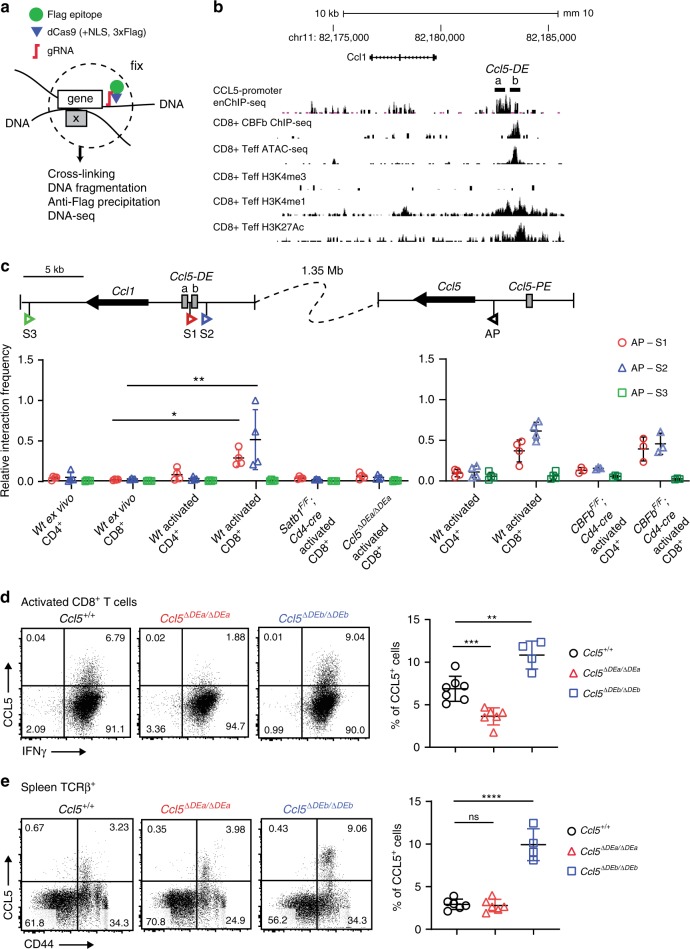
Identification of the *Ccl5 distal enhancer* by enChIP. **a** Schematic representation of a principle of enChIP (engineered DNA-binding molecule-mediated chromatin immunoprecipitation.). Briefly, dCas9 (dead Cas9)-tagged with Flag epitopes is guided to a specific genome region of interest by a single gRNA (guide RNA). After fixation to capture the chromatin around the genome region of interest, regular ChIP using an anti-Flag antibody followed by sequencing enabled us to identify interacting genomic regions. **b** An enChIP-seq track in a stimulated 2B4 mouse T-cell line expressing CCL5 and a CBFβ ChIP-seq track in CD8^+^ T cells are shown with ATAC-seq tracks and histone mark ChIP-seq tracks. Peaks of enChIP and CBFβ ChIP-seq in the *Ccl5-DE* region are shown as *Ccl5-DEa* and *Ccl5-DEb*, respectively. **c** The 3C assay was performed to validate the interaction between the *Ccl5* promoter and *Ccl5-DE*. Schematic representation (top) indicates the positions of primers (S1, S2, and S3) and an anchor primer (AP) with arrowheads and *Ccl1* and *Ccl5* with thick arrows. Gray boxes indicate *Ccl5* enhancer elements. 3C assays using these three pairs of primers were performed on the cell subsets indicated. Error bars indicate Mean ± SD and each dot represents one replicate examined over at least two independent experiments. Statistical significance is measured via unpaired two-tailed Student’s *t* tests and is presented as follows: *p < 0.05, **p < 0.01. Representative dot plots show CCL5 expression in activated CD8^+^ T cells cultured in Tc conditions for 5 days (**d**) and in TCRβ^+^ splenocytes (**e**) of mice with their genotypes indicated. In the *Ccl5*^*ΔDEa*^ and *Ccl5*^*ΔDEb*^ alleles, a 512-bp core region of the *Ccl5-DEa* and a 399-bp region of the CBFβ ChIP-seq peak were deleted, respectively, by Crispr/Cas9 genome editing. Graphs on the right are summaries of the frequencies of CCL5^+^ cells. Error bars indicate Mean ± SD and each dot represents a mouse examined over at least two independent experiments. Statistical significance is measured via unpaired two-tailed Student’s *t* tests and is presented as follows: **p < 0.01, ***p < 0.001, ****p < 0.0001, ns nonsignificant. Source data are provided as a Source Data file.

Our results predict three features of the putative *Ccl5-DE*: (1) it is located outside of the 140 kb region inserted in the *Ccl5-GFP* BAC Tg construct, (2) it should be associated with RUNX/CBFβ complexes, and (3) it should interact with the *Ccl5* promoter in activated T cells. In order to screen genomic regions that interact with the *Ccl5* promoter in an unbiased manner, we utilized a technology designated as enChIP (engineered DNA-binding molecule-mediated chromatin immunoprecipitation), which enabled us to target an epitope-tagged dCas9 (dead Cas9 lacking endonuclease activity) to genomic regions of interest (the *Ccl5* promoter in our case) via a single-guide RNA (gRNA) (Fig. 4a and “Methods”). A regular chromatin immunoprecipitation (ChIP) with an anti-epitope antibody after fixation, followed by sequencing, should detect interactions between genomic regions. We chose T-cell line 2B4 because it upregulated CCL5 expression after PMA/ionomycin stimulation; and selected a gRNA at 400 bp upstream from the *Ccl5* TSS that did not interfere with *Ccl5* promoter activity after stimulation (Supplementary Fig. 4a). After establishing a 2B4 subline expressing 3xFlag-dCas9 and gRNA using retroviral vector expression systems, we performed enChIP-seq and detected 34 genomic regions on chromosome 11 that may interact with the *Ccl5* promoter (Supplementary Fig. 4b). In order to narrow down candidates for the *Ccl5-DE* enhancer, we focused on enChIP-seq peaks that were co-bound by RUNX/CBFβ complexes. Of the 17 enChIP peaks that were near RUNX ChIP-seq peaks on chromosome 11, the closest enChIP peak to the *Ccl5* promoter was located 1.35 Mb downstream (Supplementary Fig. 4b). Interestingly, this region corresponded to one of four regions that interacted with the *Ccl5* locus as detected by an Hi-C approach using Th1 cells (Supplementary Fig. 4b), and was close to another CC chemokine gene cluster (*Ccl1/2/7/8/11/12*) (Supplementary Fig. 4b). In addition, epigenetic characteristics of the functional enhancers, such as open chromatin structures (as detected by ATAC-seq) and an accumulation of H3K4me1 and H3K27Ac enhancer-related histone modifications (as detected by ChIP-seq), were observed in this region (Fig. 4b), prompting us to investigate the interaction between this region and the *Ccl5* promoter in primary T cells using a Chromatin Confirmation Capture (3C) assay^31^. We designed the anchor primer (AP) in a region near the *Ccl5* promoter and two primers (S1 and S2) in regions near the potential *Ccl5-DE*, as well as an additional primer (S3) as a negative control (Fig. 4c). AP-S1 and AP-S2 primer pairs, but not the AP-S3 pair, produced significant amplicon signals (sequences confirmed) specifically in activated CD8^+^ T cells, while there were only weak signals from activated CD4^+^ T cells and ex vivo unstimulated T cells (Fig. 4c left panel). Therefore, our 3C assay confirmed that a long-range chromatin interaction between the *Ccl5* promoter and the *Ccl5-DE* candidate region is formed specifically in primary activated T cells in vivo, in which the distal enhancer exhibits its activity.

The locations of the enChIP-seq peak and the CBFβ ChIP-seq peak, which we referred to as *Ccl5-DEa* and *Ccl5-DEb* elements respectively, differ slightly (Fig. 4b). While the *Ccl5-DEa* lacks RSS, there are two RSSs within the *Ccl5-DEb* (Supplementary Fig. 4c). In order to examine functions of these two elements, we designed gRNAs to separately remove them by Crispr/Cas9 genome editing to investigate their functions. Upon the removal of *Ccl5-DEa (Ccl5*^*ΔDEa/ΔDEa*^ mice*)*, *Ccl5* expression in activated CD8^+^ T cells was significantly reduced (Fig. 4d), while its expression in CD44^+^ T cells during the steady state was unaffected (Fig. 4e). In contrast, *Ccl5* expression in both activated CD8^+^ and CD44^+^ memory T cells was increased by the loss of *Ccl5-DEb* (*Ccl5*^*ΔDEb/ΔDEb*^ mice) (Fig. 4d, e). These observations confirm that *Ccl5-DE* is composed of two distinct elements: one (*Ccl5-DEa*) acting as an enhancer for *Ccl5* induction in activated T cells and the other (*Ccl5-DEb*) serving as a platform for RUNX/CBFβ-associated negative regulation. Since the main enhancer activity is embedded within the *DEa* element, we hereafter refer to *Ccl5-DEa* as just *Ccl5-DE* and refer to *Ccl5*^*ΔDEa/ΔDEa*^ mice as *Ccl5*^*ΔDE/ΔDE*^ mice. There were no changes in CC chemokine expression on chromosome 11 by the removal of *Ccl5-DE*, other than CCL5 in CD8^+^ T cells 5 days after activation (Supplementary Fig. 4d), indicating that the activity of *Ccl5-DE* in activated states is restricted to the regulation of *Ccl5* at far distance.

**Fig. 5:**
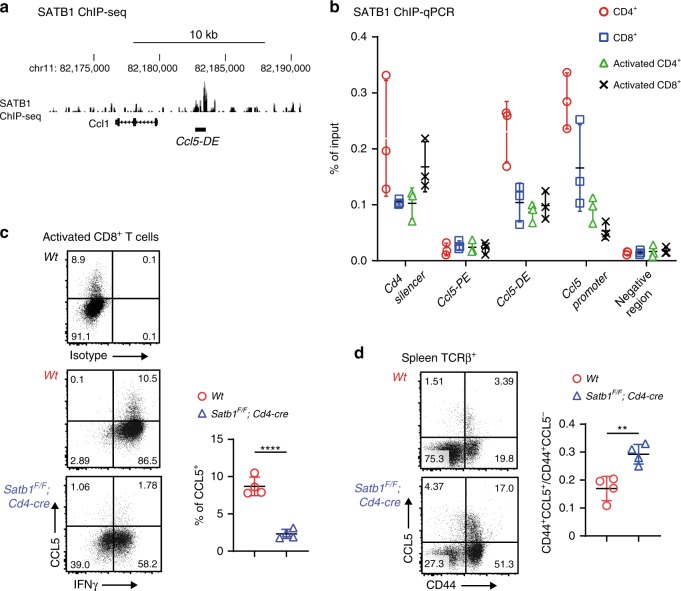
*Ccl5distal enhancer* (*DE*) regulates *Ccl5* induction in activated T cells by the help of SATB1. **a** An SATB1 ChIP-seq track in peripheral CD4^+^ T cells using GSE90635 around *Ccl5-DE*. **b** Summary of three SATB1 ChIP-qPCRs using spleen and activated CD4^+^ and CD8^+^ T cells from three mice per group. The *Cd4* silencer region was included as a positive control for SATB1 binding. Error bars indicate Mean ± SD and each dot represents a mouse examined over at least two independent experiments. **c** Representative dot plots showing CCL5 and INFγ expression in control and SATB1-deficient activated CD8^+^ T cells at 5 days after in vitro simulation with a summary graph. Error bars indicate Mean ± SD and each dot represents a mouse examined over at least two independent experiments. Statistical significance is measured via unpaired two-tailed Student’s *t* tests and is presented as follows: ****p < 0.0001. **d** Dot plots showing CCL5 expression in TCRβ^+^ T cells of *Wt* and *Satb1*^*F/F*^*;Cd4-cre* mice. One representative plot of multiple independent experiments. Numbers in the dot plots indicate the percentage of cells in each quadrant. Error bars indicate Mean ± SD and each dot represents a mouse. Statistical significance is measured via unpaired two-tailed Student’s *t* tests and is presented as follows: ****p < 0.0001, **p < 0.01. Source data are provided as a Source Data file.

Next, we investigated how such a long-range chromatin looping between the *Ccl5* promoter and *Ccl5-DE* is formed. Several nuclear proteins including CTCF, cohesin, and lamin have been shown to be involved in the formation of a long-range chromatin interaction^32^. Special AT-rich binding protein (SATB1) is another protein that has been previously characterized as a genome organizer^33,34^. SATB1 regulates gene expression by folding chromatin into loops, bringing distal genomic loci into close proximity^35,36^, and interacting with RUNX/CBFβ complexes^37^. SATB1 ChIP-seq in CD4^+^ T cells detected an SATB1 association in the *Ccl5-DE* region (Fig. 5a), and ChIP-qPCR detected an SATB1 association with *Ccl5-DE*, but not with *Ccl5-PE*, in CD8^+^ T cells (Fig. 5b). In order to examine physiological roles that SATB1 plays in *Ccl5* regulation, we stimulated SATB1-deficient CD8^+^ T cells prepared from *Satb1*^*F/F*^*;Cd4-cre* mice and found that the percentage of *Ccl5*-expressing cells had decreased by fivefold to that in control cells because of the loss of SATB1, while IFNγ expression was similar in the two cells (Fig. 5c). In line with this finding, DNA looping between the *Ccl5* promoter and *Ccl5-DE* was not detected in SATB1-deficient activated CD8^+^ T cells (Fig. 4c). On the other hand, CBFβ deficiency did not affect the looping (Fig. 4c, right panel). It is noteworthy that this long-range DNA looping formation depended upon *Ccl5-DE* (Fig. 4c). Taken together, these observations indicate that SATB1 plays an essential role in inducing *Ccl5* upon CD8^+^ T-cell activation and that an SATB1-mediated long-range DNA interaction between *Ccl5* promoter and the *Ccl5-DE* probably underlies *Ccl5* induction in these cells. Homeostatic CCL5 expression in CD44^+^ CD8^+^ T cells was rather increased from 0.17 to 0.28 (the ratio of CCL5^+^CD44^+^ divided by CCL5^−^CD44^+^) due to the SATB1-deficiency (Fig. 5d), suggesting that the short-range interaction between the *Ccl5* promoter and *Ccl5-PE* is likely to be independent of SATB1.

### Homeostatic CCL5 regulates properties of local immune cells

**Fig. 6:**
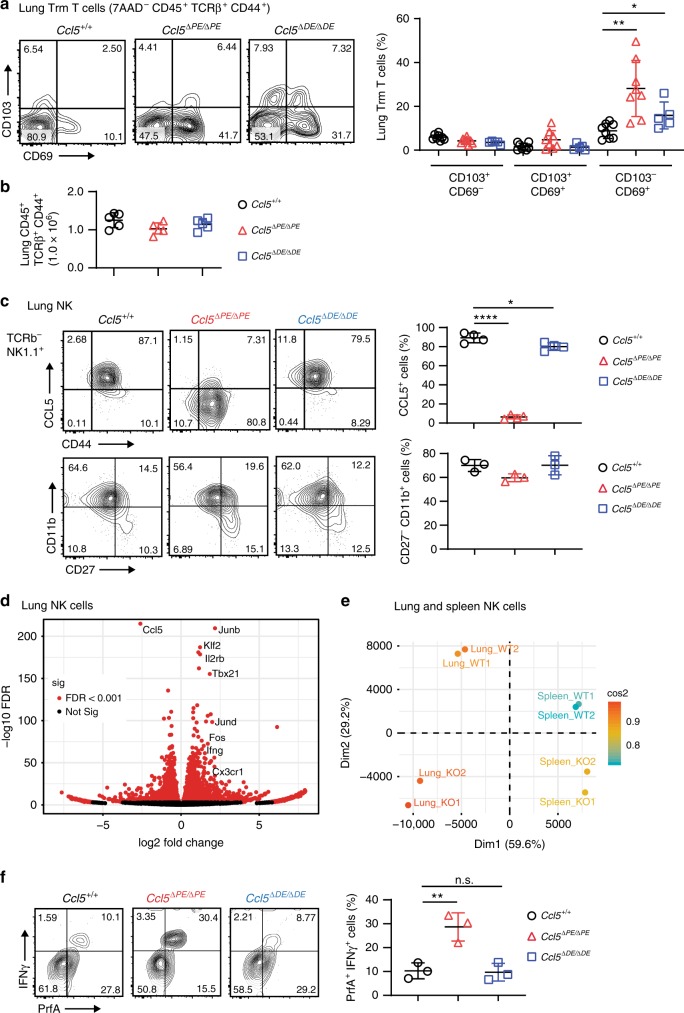
Activation status of lung Trm T- and NK-cell components are affected by the loss of homeostatic CCL5 expression. **a** Contour plots show CD69 and CD103 expression in lung Trm T cells, defined as CD45^+^ NK1.1^−^ TCRβ^+^ CD44^+^ cells, of mice of the indicated genotypes. Error bars indicate Mean ± SD and each dot represents a mouse examined over at least two independent experiments. Statistical significance is measured via unpaired two-tailed Student’s *t* tests and is presented as follows: *p < 0.05, **p < 0.01. **b** The summary graph gives a summary of the number of Trm T cells. Error bars indicate Mean ± SD and each dot represents a mouse examined over at least two independent experiments. **c** Contour plots show CCL5 expression (upper panel) and developmental status indicated by CD11b and CD27 expression (bottom panel) in the lung NK cells of mice of the indicated genotypes. Error bars indicate Mean ± SD and each dot represents a mouse examined over at least two independent experiments. Statistical significance is measured via unpaired two-tailed Student’s *t* tests and is presented as follows: *p < 0.05, ****p < 0.0001. **d** Volcano plot of RNA-seq by using lung NK cells. Selected genes are indicated by text labels. **e** Principal component analysis (PCA) of RNA-seq by using NK cells sorted from lung and spleen of mice with the indicated genotypes (KO indicates *Ccl5*^*ΔPE/ΔPE*^ mice). **f** Purified NK cells were cocultured with B16-F10 for overnight before intracellular staining with IFNγ and perforin to examine NK cytotoxicity. NK cells were defined as CD45^+^NK1.1^+^. Error bars indicate Mean ± SD and each dot represents a mouse examined over at least two independent experiments. Statistical significance is measured via unpaired two-tailed Student’s *t* tests and is presented as follows: *p < 0.05, **p < 0.01, ****p < 0.0001, ns nonsignificant. Source data are provided as a Source Data file.

Since we have established *Ccl5-PE* and *-DE* deficient mouse lines that can provide the specific loss of homeostatic and activation-induced CCL5 expression, respectively, we examined the physiological relevance of CCL5 expression at two distinct phases. Even though Trm T-cell populations in peripheral nonimmune tissues such as human vagina and mouse skins have been already reported to be reduced by the treatment of anti-CCL5 antibody^9,10^, it is not known whether CCL5 plays the similar functions in other tissues such as lungs and which homeostatic or induced CCL5 expression is important to maintain tissue-resident cells. Interestingly, the total numbers of lung Trm T cells identified as CD45^+^TCRβ^+^CD44^+^ were similar in the three mouse lines of WT, *Ccl5*^*ΔPE/ΔPE*^, and *Ccl5*^*ΔDE/ΔDE*^. However, the proportion of CD44^+^ T cells with the CD69^+^ phenotype had significantly increased in *Ccl5*^*ΔPE/ΔPE*^ and to a lesser extent in *Ccl5*^*ΔDE/ΔDE*^ mice (Fig. 6a, b). The specific increase of lung CD69^+^ Trm T-cell subset by the loss of CCL5 suggests that Trm T-cell populations in the mouse lung are maintained in a manner different from that in the human vagina and mouse skins. Furthermore, as intracellular signaling from CD69 has recently been shown to affect the functional signatures of Trm T cells^38^, the specific increase of CD69^+^ Trm T-cell subset in the lung microenvironment by the loss of CCL5 might affect the total functionality of the lung Trm T-cell population.

Compared with CCL5 expression only in a limited percentage of T cells, most of the NK cells express CCL5 (Figs. 3c and 6c) and reside in both immune tissues (such as spleen) and nonimmune tissues (such as lung), at the latter they play important roles in regulating local immunity. Lung NK cells also depend on the *Ccl5-PE* for the expression of CCL5 during the steady state. (Fig. 6c, upper panel) and appear to be normally matured as examined by the staining of CD11b and CD27 (Fig. 6c, bottom panel). We thereby next compared functional status of lung NK cells in the absence of *Ccl5-PE* by RNA-seq. As expected, CCL5 was the most significantly downregulated gene in lung NK cells of *Ccl5-PE* knockout mice (Fig. 6d). In the lung NK cells, around two-thirds of total mRNAs were either increased or decreased by the absence of *Ccl5-PE*, indicating that the lack of homeostatic CCL5 expression dramatically changes gene expression signatures in lung NK cells (Fig. 6d). The principle component assay of RNA-seq results obtained from lung and spleen NK cells indicated that splenic NK cells are distinct from lung NK cells (Fig. 6e). This is consistent with previous reports that NK cells show organ-specific mRNA signatures^39,40^. Interestingly, our results further suggest that the difference of lung NK cells between *Wt* and *Ccl5-PE* knockout mice is more significant than those in spleen NK cells (Fig. 6e). Especially, genes known to regulate NK-cell proliferation and activation such as *Junb*/*Jund*, *Tbx21* (T-bet), *Il2rb*, and *Fos* were significantly upregulated in lung NK cells of *Ccl5-PE* knockout mice (Fig. 6d). These findings not only show that the reduction of homeotic CCL5 levels has significant impacts on NK-cell properties, but also indicate that tissue NK cells in *Ccl5*^*ΔPE/ΔPE*^ mice are likely to possess more potent activities of NK cells. Thus, our results suggest that CCL5 could serve as a regulatory factor that suppresses cell functionality of NK cells.

### Homeostatic CCL5 inversely correlates with cancer immunity

Recent studies have shown that the majority of the tumor infiltrating lymphocytes (TILs) in several solid tumors are comprised of a variety of tissue-resident lymphoid cells, including Trm T and NK cells, rather than blood-borne circulating lymphocytes^41,42^. Among the various proteins secreted by TILs, host CCL5 is receiving much attention as a key anticancer molecule in mediating proper tumor immunity^11,19^. Interestingly, recent studies using CCL5 knockout mice showed that the absence of host CCL5 enhances antitumor immunity of neutrophils and T cells in breast cancer models^20,21^. Given altered properties of NK cells toward more stimulated states in the absence of *Ccl5-PE*, we examined activation status of NK cells after stimulation by cancer cells in vitro. NK cells of *Ccl5*^*ΔPE/ΔPE*^ mice were more quickly activated as indicated by higher expression of IFNγ and perforin (Fig. 6f), supporting our observation by RNA-seq experiments of ex vivo lung NK cells (Fig. 6d).

**Fig. 7:**
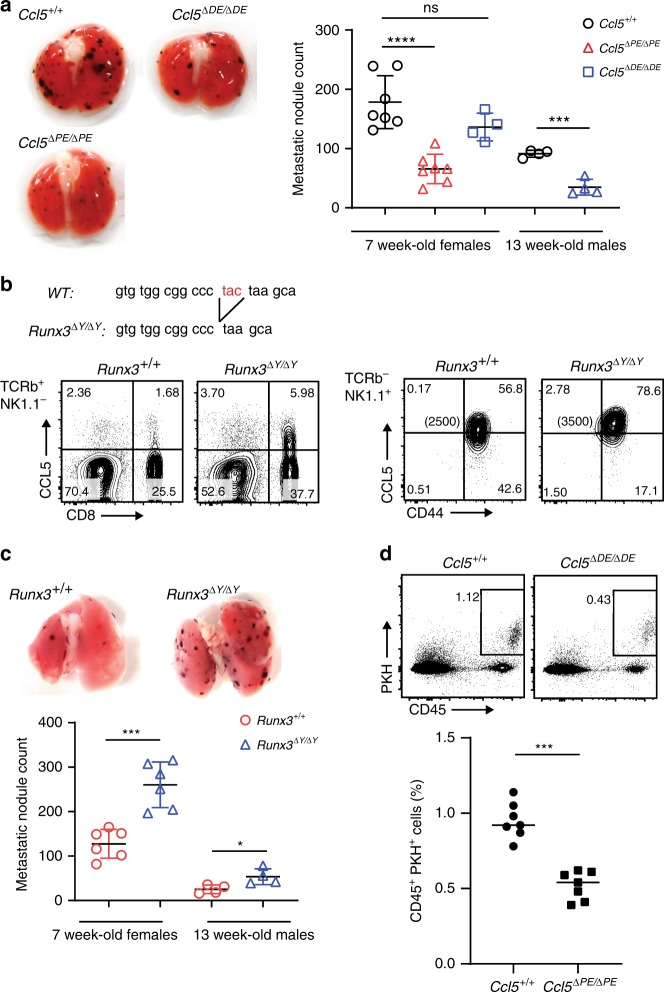
Homeostatic expression of host CCL5 is important for B16-F10 metastasis. **a** B16-F10 melanoma cells were intravenously injected into *Ccl5*^*+/+*^*, Ccl5*^*ΔPE/ΔPE*^, and *Ccl5*^*ΔDE/ΔDE*^ mice. Representative macroscopic images of lungs 14 days after injection are shown. The summary graph on the right shows the numbers of metastatic nodules. Error bars indicate Mean ± SD and each dot represents a mouse examined over at least two independent experiments. Statistical significance is measured via unpaired two-tailed Student’s *t* tests and is presented as follows: ***p < 0.001, ****p < 0.0001, ns nonsignificant. **b** Sequences of the *Runx3*^*ΔY*^ allele confirmed the removal of the last tyrosine residue from the RUNX3 protein in *Runx3*^*ΔY/ΔY*^ mice. The TAC codon for tyrosine was changed to a TAA stop codon (upper). Contour plots showing CCL5 expression in T and NK cells of *Runx3*^*+/+*^ and *Runx3*^*ΔY/ΔY*^ mice (bottom). One representative plot of four independent mice are shown. Numbers in the plots indicate the percentage of cells in each quadrant, and numbers in parentheses indicate the mean fluorescent intensity of CCL5. **c** The result of B16-F10 melanoma lung metastasis in *Runx3*^*+/+*^ and *Runx3*^*ΔY/ΔY*^ mice is shown as in **a**. Error bars indicate Mean ± SD and each dot represents a mouse examined over at least two independent experiments. Statistical significance is measured via unpaired two-tailed Student’s *t* tests and is presented as follows: *p < 0.05, ***p < 0.001. **d** The result of YAC-1 cancer model. PKH67-labeled mouse lymphoma YAC-1 was injected i.v. into *Ccl5*^*+/+*^ and *Ccl5*^*ΔPE/ΔPE*^ mice and survived cancer cells in lungs were counted. Error bars indicate Mean ± SD and each dot represents a mouse examined over at least two independent experiments. Statistical significance is measured via unpaired two-tailed Student’s *t* tests and is presented as follows: ∗∗∗*p* < 0.001. Source data are provided as a Source Data file.

We next examined whether reduced homeostatic CCL5 expression has physiological impact on lung metastasis model of B16-F10 melanoma, in which NK cells are known to play a major role in eradicating cancer cells. Fourteen days after an intravenous injection of B16-F10 cells into the mice, the number of metastatic lesions was counted (Fig. 7a). The *Ccl5*^*ΔPE/ΔPE*^ group exhibited a significant reduction in the number of metastatic lesions compared with the control *Ccl5*^*+/+*^ and *Ccl5*^*ΔDE/ΔDE*^ groups, indicating that antimetastatic cancer immunity is enhanced by reduced homeostatic *Ccl5* expression in host cells in the B16-F10 melanoma lung metastasis model. In order to examine the effects of increased *Ccl5* expression on B16-F10 metastasis, we generated a *Runx3* mutant mice lacking the last tyrosine (Y) residue, referred to as *Runx3*^*ΔY/ΔY*^ mice (Fig. 7b). Similarly, to mice lacking the C-terminal VWRPY penta-peptide motif (*Runx3*^*ΔV/ΔV*^ mice), the lack of the last Y residue alone increased the percentage of CCL5-expressing CD44^+^ CD8^+^ T cells and the expression levels of CCL5 in NK cells. Importantly, *Runx3*^*ΔY/ΔY*^ mice exhibited increased numbers of metastatic lung lesions compared with control *Runx3*^*+/+*^ mice (Fig. 7c). Also, cancer cells were more rapidly eliminated by NK cells in another cancer model using YAC-1 mouse lymphoma (Fig. 7d). These findings suggest that homeostatic *Ccl5* expression in host cells is beneficial for the survival of cancer cells at metastatic sites, indicating that homeostatic host CCL5 is a procancer molecule in mouse cancer models.

To directly examine the functional state of tumor infiltrated NK cells, we utilized B16-F10 skin growth model in which we can readily purify NK cells from tumor lesions. Consistent with the lung metastasis model, tumor lesions were smaller in *Ccl5*^*ΔPE/ΔPE*^ mice and were larger in *Runx3*^*ΔY/ΔY*^ mice, compared with the control mice (Supplementary Fig. 5a). As expected, tumor infiltrated NK cells in *Ccl5*^*ΔPE/ΔPE*^ and in *Runx3*^*ΔY/ΔY*^ mice exhibited an increase and decrease in CD107a (LAMP-1) expression, respectively, which is indicative of lytic granule releases and is widely used as a functional marker of NK cell activation (Supplementary Fig. 5b), although NK cells in nontumor tissues in all three lines similarly did not express CD107a. In order to further investigate the involvement of the CCL5–CCR5 axis in NK-mediated B16-F10 killing, we performed an in vitro NK-killing assay in which NK cells and B16-F10 were cocultured for 4 h. We observed that blocking the CCL5–CCR5 axis using an anti-CCL5 antibody or CCR5 inhibitors resulted in an increase in apoptotic B16-F10 cells (Supplementary Fig. 5c). The increased apoptotic B16-F10 cells by blocking the CCL5–CCR5 axis support a model that enhanced tumor immunity in *CCL5-PE* knockout mice is likely to reflect the direct effect of the activated properties of NK cells rather than the secondary effects by another cell types. Collectively, our results are supportive to the hypothesis that the host CCL5 might facilitate cancer cells into evading the host’s antitumor immune responses by suppressing at least the activation status of NK cells, at least in the B16-F10 and YAC-1 metastatic models.

## Discussion

CCL5 is an important inflammatory chemokine that is induced at the late phase of inflammation, but recent studies have highlighted its other role in maintaining Trm T cells during the steady state. In the current study, we show concrete genetic evidence that *Ccl5* expression during these distinct phases, inflammation vs. homeostasis, is regulated by two distinct enhancers, providing insights into *Ccl5* regulation and advancing the previous works that have mainly focused on how the *Ccl5* promoter is regulated. Our results demonstrate that these *Ccl5* enhancers are finely regulated by RUNX/CBFβ complexes and SATB1 chromatin organizer, unraveling that sophisticated mechanisms are in work to control *Ccl5* expression. Especially, RUNX-mediated repression not only fine-tunes the kinetics of *Ccl5* expression but also limits the cell-types expressing *Ccl5* during activation. The significance of RUNX-mediated *Ccl5* repression is highlighted by the restoration of allergic inflammation by the removal of CCR5 in *Cbfb*^*F/F*^;*Cd4-Cre* mice (Seo, manuscript in preparation).

Our results suggest that the mechanisms by which RUNX/CBFβ antagonizes two *Ccl5* enhancers presumably differ. While the *Ccl5-DE* is suppressed by indirect association of the RUNX/CBFβ via the adjacent repressive element, the *Ccl5-PE* could be antagonized by direct recruitment of RUNX/CBFβ via RSSs embedded within *Ccl5-PE*. On the other hand, it is unclear how RUNX-mediated repression is canceled in *Ccl5*-expressing cells. The transcription factor FLI-1 was shown to induce CCL5 expression in endothelial cells by binding to the *Ccl5* promoter^43^, and Klf13 transcription factor was shown to be involved in *Ccl5* activation by binding to the promoter during inflammation^44,45^. Based on the presence of binding sites for IRF-1, NFkB, AP-1, C/EBP, and Ets-1 within the *Ccl5* promoter, these factors have been proposed as potential regulators that can activate *Ccl5* upon exposure to inflammatory signals, such as LPS, TNF, or IFNγ^46–48^. However, little is known about how these factors regulate *Ccl5* in detail. The identification of functional enhancers in our current study provides clues to understand how these potential regulators function to activate *Ccl5*. Indeed, the public ChIP-seq database revealed that BCL11B, TCF1, P300, JUND, and IRF4 are associated with *Ccl5-PE and Ccl5-DE* in T-cell subsets. It is important to ascertain how these possible active regulators counteract RUNX-mediated repression in future studies.

To our surprise, *Ccl5-DE* locates 1.35 Mb away from *Ccl5*, and there are bunches of other genes between *Ccl5-DE* and *Ccl5*. Previous studies have identified a few of far distance enhancers, such as an MYC superenhancer^49^ and Class I olfactory receptor enhancer^50^ acting from 1.7 or 1.3 Mb, respectively. Our 3C assay shows that SATB1 plays an important role in mediating the long-distance interaction between the *Ccl5-DE* and the *Ccl5* promoter. SATB1 functions as a genome organizer by providing the SATB1 nuclear architectural framework onto which chromatin is anchored by tethering specialized genomic regions of chromatin known as base-unpairing regions^35,36,51^. Previous studies^37,52^ showed that SATB1 binds to enhancers and a silencer, but understanding of the exact mechanisms by which SATB1 mediates the long-distance interaction between the *Ccl5-DE* and *Ccl5* promoter waits for further experiments.

Previous studies showed that CCL5 plays an important role in the homeostatic maintenance of Trm T cells in the mouse skin and human vaginal tissues by experiments using neutralizing anti-CCL5 antibodies. Even though it was assumed that homeostatic CCL5 expression at the local tissues is responsible for homeostasis of Trm T cells, the definitive conclusion has been waiting for studies using genetic tools that enable to distinguish roles of homeostatic and inducible CCL5 expression. By generating *Ccl5-PE* and *Ccl5-DE* knockout mouse lines, we provide such genetic tools which can be used to answer those questions in the future experiments. In addition, we observe a proportional increase of CD44^+^ CD69^+^ Trm T subset in the lungs by the loss of CCL5 expression. Currently, the precise function of CD44^+^ CD103^−^CD69^+^ Trm T-cell subset in lungs remains unclear, even though it is hypothesized that CD69^+^ Trm T-cell subsets provide more robust inflammatory responses based on association of CD69 with activated status. Our transcriptome analyses show that NK cells also acquire activated state upon reduction of tissue CCL5 levels, unraveling a previously unidentified role of CCL5 in conditioning functional properties of immune cells at nonimmune tissues. It will be of interest to characterize the lung Trm subset and investigate how immune responses at the lung are affected in *Ccl5*-*PE* knockout mice.

Previous studies have shown that host CCL5 can work as both pro- and anticancer molecule, generating a controversy in its role in cancer immunity. Our results provide supporting evidence to the model for the procancer action of CCL5. Contrary to previous studies using anti-CCL5 antibody treatment or CCL5 germline knockout mouse to inactivate all host CCL5, the mouse models we generated are unique and lack specifically either homeostatic or inflammatory *Ccl5* expression. Our study demonstrates that host responses against metastatic cancer are mainly regulated by homeostatic *Ccl5* expression in local tissue-resident immune cells. Mechanistically, we provide evidence that homeostatic CCL5 could condition the functional status of local immune cells such as Trm T and NK cells. Since the main producers of CCL5 in steady states are in fact Trm T and NK cells themselves, it is possible that the autocrine signaling of CCL5 might be responsible for the observed changes in their functional status. However, in cancer microenvironments in which other types of immune cells, such as DCs and Tregs, are present, it is possible that CCL5 secreted from those cells also affects the functional status of Trm T and NK cells, in addition to autocrine signaling.

Our results do not formally exclude another important possibility that CCL5 might directly affect the survival of cancer cells. Previously, CCL5 was shown to induce changes in the cellular properties of CCL5-bound T cells, such as proliferation rate and activation status, by receptor-mediated intracellular signaling^53^. In addition, another report showed that CCL5 can function as a pleiotropic factor to directly affect the differentiation of hematopoietic stem cells^54^. In our in vitro coculture system, a CCL5–CCR5 axis blockade rapidly increases the percentage of apoptotic B16-F10 cells within a short time window. Although it remains possible that CCL5 inhibition alters the functional states of NK cells to dampen cytolytic activity within a few hours, which is consistent with our in vivo results, our results suggest a possibility that CCL5 acts directly on B16-B10 cells to support their survival. In this scenario, reduction of CCL5 levels in the cancer microenvironments of *Ccl5*^*ΔPE/ΔPE*^ mice might restrict the availability of rejuvenating CCL5 to cancer cells.

Interestingly, a study utilizing CCR5-Δ32 polymorphism, which results in the expression of nonfunctional CCR5, reported an association of such mutation with worse outcomes of melanoma after immunotherapy^55^, which suggested an anticancer role of CCR5 signaling. Since the CCR5-Δ32 polymorphism is retained in both immune and cancer cells in patients, it is difficult to precisely attribute the contribution of CCR5 signaling in this case. It is equally possible that CCR5-Δ32 polymorphism of immune cells weakens their own cytotoxic activity or that CCR5-Δ32 polymorphism of cancer cells enhance their own survival and growth. Identifying the ligands for CCR5 signaling in this case might help to interpret this phenomenon. On the other hand, the effectiveness of a CCR5 inhibitor (Maraviloc) in a clinical trial of cancer patients indicates that inhibition of CCR5 signaling might be a promising target for cancer immunotherapy^22^. Further studies that address how CCR5 signaling creates both anti- and procancer microenvironments by acting on host immune cells, as well as cancer cells, will provide useful insights for future drug development.

## Methods

### Mice

*Runx1*^*ΔV*^^56^, *Runx3*^*ΔV*^^57^, *Cbfβ*^*F/F*^:*Cd4-cre*^26^, and *Satb1*^*F/F*^:*Cd4-cre*^37^ mice have been reported. In this study, *Ccl5* enhancer knockout mice (*Ccl5*^*ΔPE/ΔPE*^ and *Ccl5*^*ΔDE/ΔDE*^) were generated by using two gRNAs encompassing the regions intended for the deletion. The deleted regions are as follows: *Ccl5-PE* (Chr11:83,535,110–83,535,294 in GRCm38.p4 C57BL6J) and *Ccl5-DE* (Chr11: 82,182,569–82,183,080 in GRCm38.p4 C57BL6J). In this study, *Runx3*^*ΔY/ΔY*^ was generated by using one gRNA close to the sequences encoding the last tyrosine amino acid of RUNX3 protein. gRNA sequences are provided in Supplementary Table 1. All unique mutant mouse lines generated in this study are readily available on request. All mouse lines were separately housed and bred. Mice used in this study were between 1.5 and 4 months old and were mixed sexes otherwise specified. Mice were euthanized by suffocation with CO_2_ under anesthesia or cervical dislocation. All mice were maintained in the specific pathogen free animal facility at the RIKEN IMS, and all animal procedures were in accordance with institutional guidelines for animal care and with the protocol (28-017) approved by Institutional Animal Care and Use Committee (IACUC) of RIKEN Yokohama Branch.

### Cell cultures

T cells from spleen or lymph nodes were enriched by MACS magnetic beads (Miltenyi Biotec) and were sorted by FACS Aria (BD Biosciences) before in vitro stimulation experiments. Purified T cells were cultured in custom ordered Dulbecco’s Modified Eagle Medium (D-MEM, KOHJIN BIO) supplemented with 10% fetal bovine serum (FBS) (Hyclone). For in vitro culture, 2.0 × 10^5^ cells were stimulated in 96-well plates by precoated 2 µg/ml anti-CD3e antibody (553058, BD Biosciences) with 2 µg/ml soluble anti-CD28 antibody (553295, BD Biosciences) for 2 days. Activated T cells were maintained with 40 U/ml rmIL-2 (402-ML, R&D Systems). 2B4 cell line was maintained in RPMI medium (GIBCO) supplemented with 10% FBS and B16-F10 cell line in D-MEM medium (GIBCO) supplemented with 10% FBS. All cell culture medium was supplemented with 100 U/ml penicillin and 100 µg/ml streptomycin.

### in vitro NK cytotoxicity assay

A total of 2.0 × 10^4^ B16-F10 cells were plated 1 day before the assay on a 48-well plate. NK cells were purified by the NK Cell Isolation Kit (Miltenyi Biotec) and stimulated with 200 U/ml rmIL-2 (402-ML, R&D Systems) for 24 h. Same numbers (2.0 × 10^4^) of activated NK cells were cocultured with B16-F10 cells for 4 h. In order to inhibit CCL5–CCR5 interaction, 0.5 µg/ml of Maraviroc (PZ0002, Sigma-Aldrich), 0.5 µg/ml of TAK779 (SML0911, Sigma-Aldrich) or 0.5 µg/ml anti-mCCL5 (MAB478-100, R&D Systems) antibody were added together with NK cells.

### Tumor metastasis and growth in mice

For lung metastasis model, 1.0 × 10^5^ B16-F10 melanoma cells were suspended in HBSS and injected via the tail vein. On day 14, the mice were euthanized, and the lungs were perfused with PBS via the trachea. Lung metastases were visually counted under a dissection microscope. For growth model of B16-F10 melanoma cells at the skin of host mice, 5.0 × 10^4^ B16-F10 cells were suspended in HBSS and subcutaneously injected into the right flanks of mice. Tumor volume was measured around twice per week using calipers. After 14–20 days, mice were euthanized when their tumor volumes reached 4000 mm^3^, which is 50% of the tumor volume allowed by IACUC of RIKEN Yokohama Branch. Tumor volume was calculated using the following formula: volume = (width)^2^ × length/2. For YAC-1 cancer model, PKH67 (PKH67GL, Sigma-Aldrich)-labeled 2.0 × 10^6^ YAC-1 cells injected i.v. into mice and analyzed after 6 h. Lungs were dissected and examined by flow cytometry for survived CD45^+^ PKH67^+^ YAC-1 cells. Lungs and tumors were digested using an enzyme mix containing 1 mg/ml collagenase (C5138, Sigma-Aldrich) and 200 μg/ml DNase (D5025, Sigma-Aldrich) for 20–30 min at 37 °C while rotating, followed by filtering through a 50 μm filter.

### BAC transgenic mice

GFP reporter constructs were generated by using the Quick & Easy BAC Modification Kit (Gene Bridges) according to the manufacture’s instruction. Briefly, *Ccl5* coding region from the start codon to stop codon (4741 bp) on BAC clone, B6Ng01-015L02 purchased from RIKEN BioResource Research Center, was replaced with eGFP cDNA, generating a *Ccl5-GFP* BAC reporter Tg construct. The deletion of 1201 bp Runx binding region (RBR) and mutations on two RRSs within the *Ccl5-RBR* onto the *Ccl5-GFP* BAC construct were achieved by the Counter Selection BAC Modification Kit (Gene Bridges). The resulting three BAC Tg constructs were purified and injected at Institute of Immunology Co. LTD.

### Chromatin immunoprecipitation assays

ChIP assays using anti-CBFβ^26^ and SATB1 (ab70004, Abcam) antibodies were performed as follows^58^. In brief, CD4^+^ or CD8^+^ T cells were enriched by MACS magnetic beads (Miltenyi Biotec) and crosslinked with 1.0% formaldehyde for 10 min at room temperature. The reaction was stopped by adding final 0.15 M glycine and the cells were washed extensively with PBS. Fixed cells were lysed with lysis buffer containing 0.5% NP-40 and 0.25% Triton X-100, and nuclei pellets were resuspended in sonication buffer containing 0.1% Sodium deoxycholate and 0.5% N-laurylsarcosine sodium salt. The nuclei were sonicated using XL2000 ultrasonic cell disruptor (Microson) at output level 6 for 15 s between 8 and 10 times to yield 200–300 bp fragments. Sonicated chromatin was immunoprecipitated by either 1 μg of anti-CBFβ or anti-SATB1 antibody. Captured DNA fragments were washed with RIPA buffer and purified by the ChIP DNA Clean and Concentrator Kit (Zymo Research). The primers used for the ChIP-qPCR is provided in Supplementary Table 1.

### ChIP-seq

For ChIP-seq analysis, purified DNAs from ChIP samples were used for the library construction with NEBNext ChIP-Seq Library Prep Reagent Set for Illumina (NEB) according to the manufacturer’s protocol. Libraries were quantified with the KAPA Library Quantification Kit for Illumina (KAPA Biosystems) and the quality was checked by Bioanalyzer using Agilent High Sensitivity DNA ChIP (Agilent Technologies). Sequencing was performed by genomics facility at RIKEN IMS with Illumina HiSeq2000/2500. Sequencing results were aligned to the mouse reference genome (mm10) using the bowtie2 (version 2.3.5.1) with standard parameters. Significant peaks from enChIP-seq were obtained by macs2 (version 2.2.5) and visualized on UCSC Genome browser.

### enChIP and enChIP-Seq

The enChIP-seq assay was performed on *Ccl5* promoter as follows^59^. In brief, 2B4 cell line was transduced with a retroviral plasmid (pMSCV-Flag-dCas9-ires-eGfp) encoding Flag-tagged dead Cas9 followed by IRES-eGFP. GFP^+^ 2B4 cells were sorted and were transduced again with a retroviral plasmid (pSir-hCD2) encoding the gRNA, which is driven by the U6 promoter and corresponds to the region 400 bp upstream of *Ccl5* TSS, followed by hCD2 reporter driven by the histone H4 promoter. The gRNA sequence used is 5′-GAGAGGGAGTCATCCTGGAC-3′. GFP^+^ hCD2^+^ cells were sorted and were expanded for enChIP-seq analysis with anti-Flag antibody M2 (F3165, Sigma-Aldrich). Immunoprecipitation and analysis of sequencing data are identical to those described in ChIP-seq procedures.

### Quantitative RT-PCR and RNA-seq

mRNA was purified by using the Direct-zol RNA kits (Zymo Research) according to the manufacturer’s protocol that includes on-column DNaseI treatment. Purified RNA was converted into cDNA by using the SuperScript IV First-Strand Synthesis System (Thermo Fisher) according to the manufacture’s protocol. Obtained cDNA was used as template for quantitative real-time PCR by using Power SYBR Green PCR Master Mix (Thermo Fisher) according to the manufacture’s protocol. The sequences of primers used for the qPCR are provided in Supplementary Table 1. Purified mRNAs were used for the library construction with the NEBNext Ultra II RNA Library Prep Kit for Illumina (NEB) according to the manufacturer’s protocol. Library was quantified, checked and sequenced as described for the ChIP-seq. Sequencing results were aligned to the mouse reference genome (mm10) using the HISAT2 (version 2.1.0) with standard parameters. Read counts were obtained by HTSeq (version 0.11.0) and further analyzed by a Bioconductor package EdgeR (version 3.6.0).

### Enzyme-linked immunosorbent assay and Luminex analysis

An anticytokine (or chemokine) antibody was coated to ELISA plates (Nunc Maxisorb) at 1 µg/ml in 0.1 M Na2HPO4 (pH 9.0) at 4 °C overnight. After rinsing with PBS three times, the plate was blocked with PBS containing 10% FBS at room temperature for 1 h. Blocked plates were washed three times in PBS with 0.05% Tween-20. Standard cytokines and chemokines as well as sample supernatants were diluted in PBS with 10% FBS and 0.05% Tween-20 and incubated for 4 °C overnight. After washing three times in PBS with 0.05% Tween-20, a biotinylated antibody against the target cytokine (chemokine) was added at 1 µg/ml in PBS with 10% FBS and 0.05% Tween-20 at room temperature for 1 h. After washing three times in PBS with 0.05% Tween-20, streptavidin-HRP conjugate (Bethyl Laboratories, INC) was incubated in PBS with 10% FBS and 0.05% Tween-20 at room temperature for 30 min. Plates were further washed three times in PBS with 0.05% Tween-20 and three times in water. TMB substrate (Thermo Fisher Scientific) was added and incubated at room temperature for 15 min before adding the stop solution (sulfuric acid) before measuring an absorbance at 450 nm. Antibodies and antigens for ELISA were included in the following assay kits from Thermo Fisher: IL-3 (AHC0832), IL-4 (16–7041–81), IFNγ (M700A), CCL3 (12–7532–82), CCL4 (MA5-23713), and CCL5 (AHC1052). For multiplex Luminex analysis, Bio-Plex ProMouse Cytokine 23-plex Assay (Bio-Rad) was used and performed by proteomics facility at RIKEN IMS.

### Flow cytometry and antibodies

Live cell suspension from splenocytes were prepared by standard procedures. Single-cell suspensions were stained with the following antibodies purchased from BD Bioscience, Biolegend, or Thermo Fisher: Annexin V (640937, Biolegend), CCL1 (PA5-47952), CCL5 (2E9/CCL5), CD4 (RM4-5), CD8α (53–6.7), CD8β (YTS156.7.7), CD11b (M1/70), CD25 (PC61), CD44 (IM7), CD45.2 (104), CD69 (H1.2F3), CD103 (M290), IFNγ (XMG1.2), NK1.1 (PK136), Gr-1 (RB6-8C5), Ter119 (Ter119), TCRβ (H57-597), TCRγδ (GL3), and CD107a (eBioH4A3), which were used at 0.5 μg/ml. For intracellular staining, cell suspensions were fixed and permeabilized at the same time by BD Perm/Fix solutions (BD Biosciences). Multicolor flow cytometry analysis was performed using a FACSCanto II (BD Biosciences), and data were analyzed using FlowJo version 10 (Tree Star) software.

### 3C assay

Sorted T cells were used for standard 3C assays^60^. Briefly, 1.0 × 10^7^ cells were fixed with 2% formaldehyde at room temperature for 10 min and final 0.2 M glycine was added to quench free formaldehyde. Crosslinked cells were lysed with ice-cold lysis buffer (10 mM Tris (pH 7.5), 10 mM NaCl, 5 mM MgCl2, 0.1 mM EGTA) with Complete Protease Inhibitor (Roche) on ice for 10 min. The nuclei were harvested and digested with 400 U of BglII (NEB) at 37 °C while shaking at 900 rpm overnight. After heat inactivation of the restriction enzyme at 65 °C for 20 min, digested DNA was ligated with 100 Weiss Unit of T4 DNA ligase (Promega) at 16 °C for 4 h. Ligated DNA was purified by adding 0.3 mg Proteinase K (Invitrogen) and incubating at 65 °C overnight. Samples were further purified by the standard phenol extraction and ethanol precipitation. As a control locus, *Gapdh* promoter region was chosen to normalize each PCR reactions. Data are presented as relative crosslinked frequency and obtained from at least two independent biological replicates with duplicates. As control experiments, no PCR products were confirmed when the ligation step was omitted. The PCR products after qPCR were sequenced to confirm proper ligation for every experiment. The sequences of primers used for the 3C assay qPCR are provided in Supplementary Table 1.

### Statistical analysis

The individual dots on the summary graphs indicate independent mice analyzed, otherwise specified. Statistical parameters are indicated in the figure legends. All bar and dot graphs show mean values ± SD. Statistical analysis was performed using unpaired two-tailed Student’s *t* test in the GraphPad Prism Version 8. The *p* value of <0.05 was considered statistically significant.

### Reporting summary

Further information on research design is available in the Nature Research Reporting Summary linked to this article.

## Supplementary information

Supplementary Information

Peer Review File

Reporting Summary

## Data Availability

The data for enChIP-seq, CBFβ ChIP-seq, and RNA-seq have been deposited in GEO database under the accession code GSE130600. Standard ATAC-seq and histone modifications ChIP-seq data were obtained as fastq files from GSE95237. SATB1 ChIP-seq data were obtained as fastq files from GSE90635. Hi-C analysis of mouse Th1 cells were obtained from GSE48262 and visualized on a web tool [http://promoter.bx.psu.edu/hi-c/].

## Source Data

Source Data
