# Supplementary material for: Runx-mediated regulation of CCL5 via antagonizing two enhancers influences immune cell function and anti-tumor immunity
Source: Nat Commun. 2020 Mar 26;11:1562. doi: 10.1038/s41467-020-15375-w (PMC7099032; doi:10.1038/s41467-020-15375-w)
Supplement: Supplementary file 1 — Supplementary Information [file 41467_2020_15375_MOESM1_ESM.pdf]

Runx-mediated regulation of CCL5 via antagonizing two enhancers influences immune cell function and anti-tumor immunity

Seo et al.

SUPPLEMENTARY INFORMATION

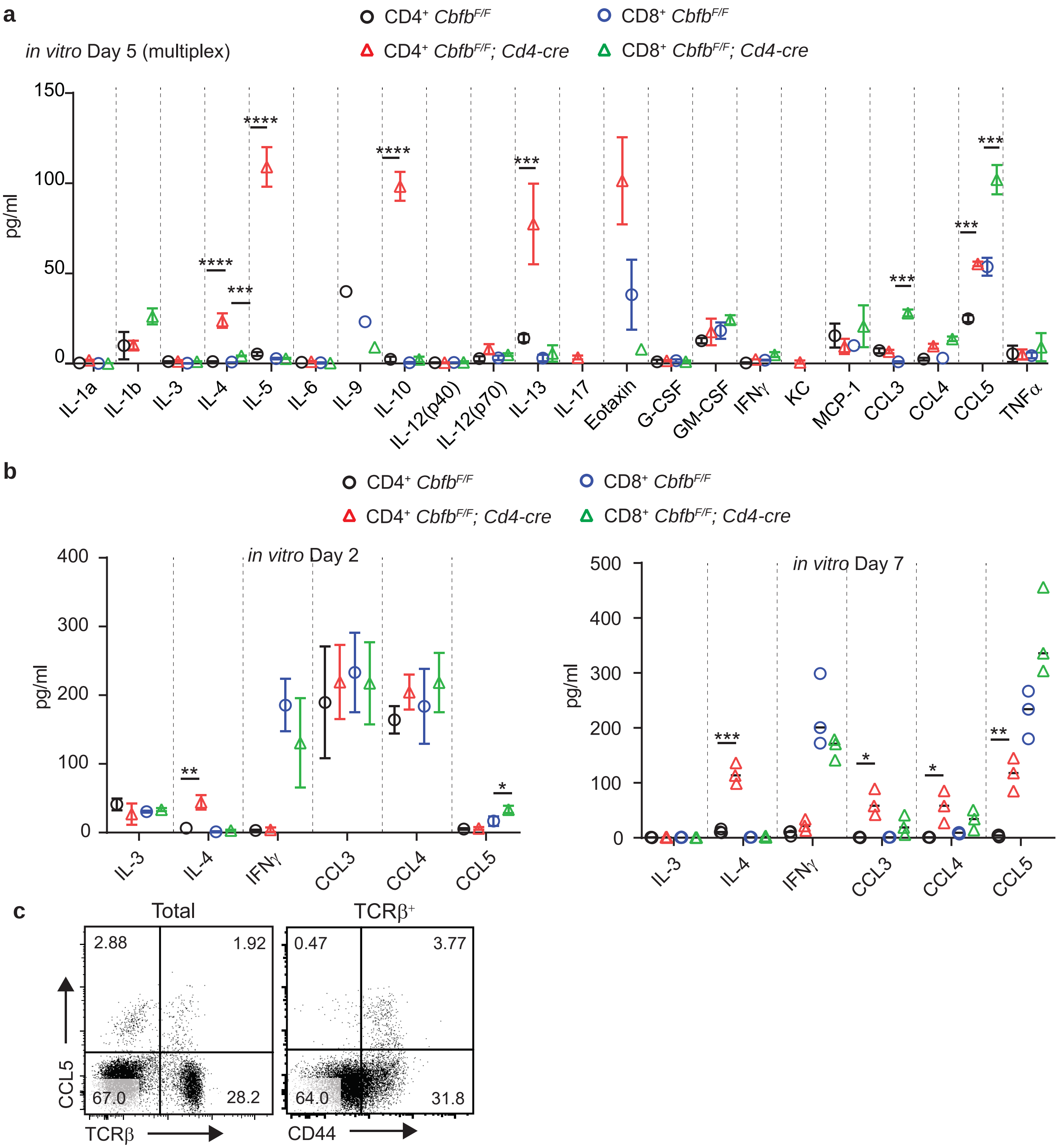

Supplementary Fig. 1.: CCL5 expression is repressed by Runx/Cbfβ complexes

a Expression profiles of 22 cytokines and chemokines were obtained from the supernatants of in vitro stimulated CD4+ and CD8+ T cells (day 5) of CbfbF/F and CbfbF/F; Cd4-Cre mice with the indicated genotypes by Luminex multiplex analysis. A summary of at least three measurements is shown.

b Expression profiles as assessed by ELISA of the selected CC chemokines CCL3, CCL4 and CCL5 and the cytokines IL-3, IL-4, and IFNγ in the supernatants of in vitro-stimulated CD4+ and CD8+ cells with indicated genotypes (days 2 and 7). Summaries of at least three independent measurements.

c Flow cytometry analysis of total splenocytes and TCRβ+ spleen T cells. One representative dot plot of over five independent experiments. Numbers in the dot plots indicate the percentage of cells in each quadrant.

a,b Error bars indicate Mean ± SD and each dot represents a mouse examined over at least two independent experiments. Statistical significance is measured via unpaired two-tailed Student's t-tests and is presented as follows: *p < 0.05, **p < 0.01, ***p < 0.001, ****p < 0.0001. Source data are provided as a Source Data file.

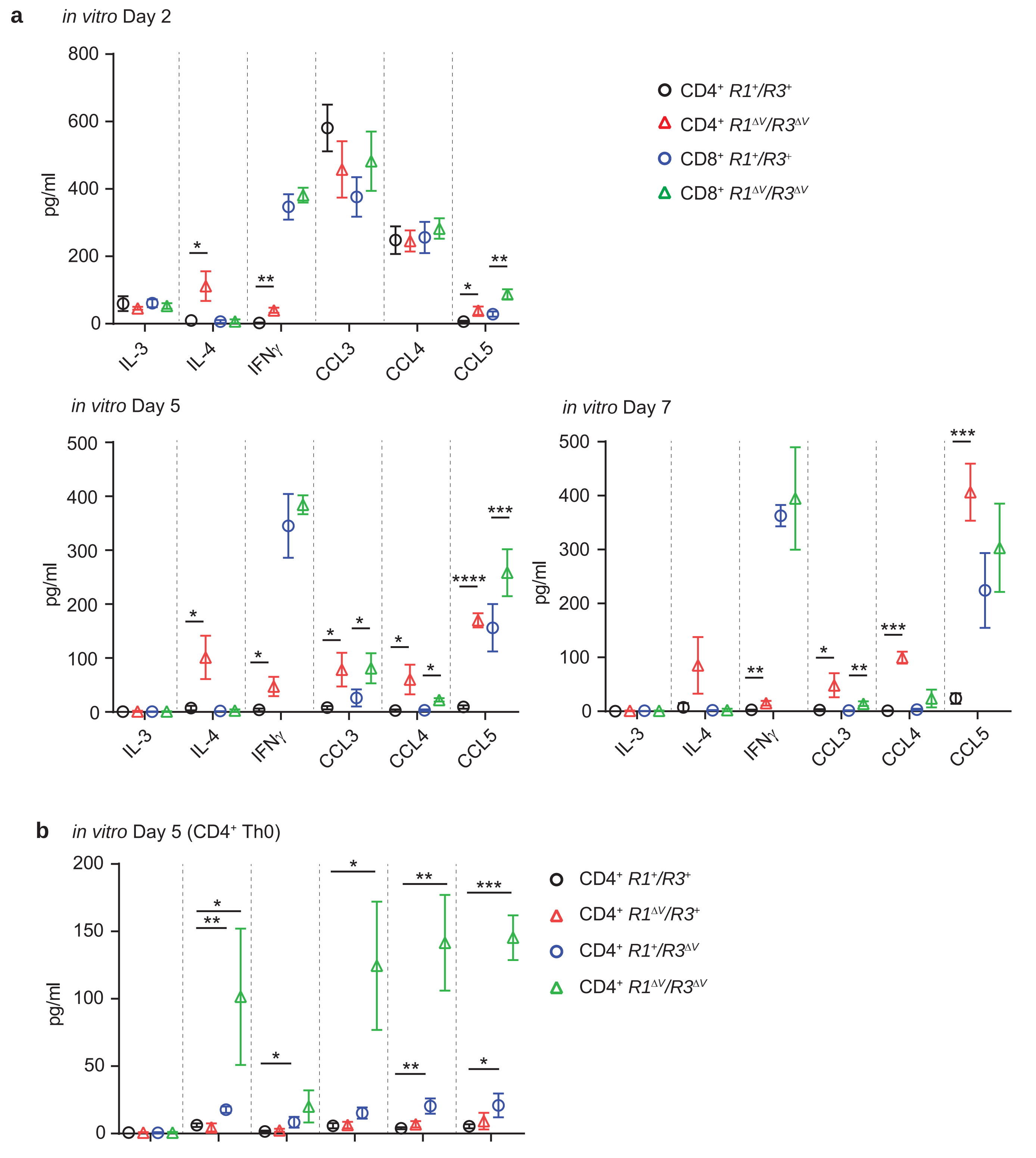

Supplementary Fig. 2.: Essential roles of the VWRPY motif in Runx proteins for CCL5 repression

a Expression profiles as assessed by ELISA of the selected CC chemokines CCL3, CCL4 and CCL5 and the cytokines IL-3, IL-4, and IFNγ in the supernatants of in vitro stimulated CD4+ and CD8+ T cells (days 2, 5, and 7) of Runx1+/+; Runx3+/+ (R1+/R3+) and Runx1ΔV/ΔV; Runx3ΔV/ΔV (R1ΔV/R3ΔV) mice.

b Expression profiles as assessed by ELISA of the selected CC chemokines, CCL3, CCL4 and CCL5 and the cytokines IL-3, IL-4, and IFNγ in the supernatants of in vitro-stimulated CD4+ T cells in Th0 culture conditions (day 5) of Runx1+/+; Runx3+/+ (R1+/R3+), Runx1ΔV/ΔV; Runx3+/+ (R1ΔV/R3+), Runx1+/+; Runx3ΔV/ΔV (R1+/R3ΔV) and Runx1ΔV/ΔV; Runx3ΔV/ΔV (R1ΔV/R3ΔV) mice.

a,b Error bars indicate Mean ± SD and each dot represents a mouse examined over at least two independent experiments. Statistical significance is measured via unpaired two-tailed Student's t-tests and is presented as follows: *p < 0.05, **p < 0.01, ***p < 0.001, ****p < 0.0001. Source data are provided as a Source Data file.

a

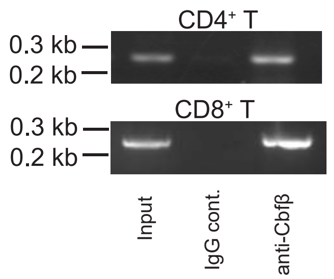

b **Ccl5-RBR:**

CTTCCAGACACATGCTACCCTGTCCTGTCCTGGCTTAGGCTTTGTTTTCTTTTCTTTCTCTTTTTGACTCT
TTTCCCAAGCTGAGCTGGCTCTCAGCCCAGTCAATGTGACGTTATGAAGCCGGCTGCAGCCAAAGAAA
CCGAAACCCCATTTCACATGGATTCCAGTGCTTTAGTCAGTCGTGGGGTTTCCCCATGACTGTGTGGGT
CAGGTTTCTCTCCTGGCCACTCCTATCATAGTGTCCGAAGTAGGGAAAATCCATAGTCTCTAGGCATCT
GGGCTCCCAGCAGAAGCCAAAAGGAAAGTCATTTCCTGCACGGCTTTAACTTTCTCCATATGCAAACTG
GAATCTGCTTCTTGATTGGATCACCTTCTGCTTCCTTACCTCTGGGACAGCAAGTAGCTGGTGGCTCAA
GCCATCTGCTTGCAGAGGGCCCTACTGACAAGGTGACAGCATCACAGTCATCACCCATGTCTCTAGTT
GCCTGCCCTCATAATAGCTCC**AACCACA**TTCAGACCAGAGGCTTGCACCCGGGCCTCCACTAGCTCTT
CTCACCTTCACCACACAGGACAGCTCGGCACACCCACTTGCTCTCTGAAGCTGGA**GACCACA**TGCGG
TGTGTTTGTGATGGGAACTGTGCTTGGGTCACAGGGGAGCCCCCACAAGGTGGTGCGAGTGTGAGG
CCTGGGGGAAGCACGTGATCAGTGTTTGTTGACAGATAATTTTAGGCCCCCTCTTCTATGAGAGGAATG
CATTTTTATTTTCTTCTTCATATTGGTTTGAGGATTCCTTTTTGTCTTTATTTATTTATTTTTTGTTTGCTTGT
TTGTTTCTTGTTTCATATCCCTCATGAAATTCCTTATCCTTTATTCTCTAGGAAAGCTTATTGCCAGAGCAT
GACCCCTCAGTTCTGCAGTCTTCCCCCCCCCCCCAGAAGGAAGTCTGATGGGGCAAGGAAGACTTGC
ATTTGAAATCAGTCAGCACTTGCGGTCTCTGTGCCTTTGGACAGATGGTTAAATCGACTCCTCGGTTTC
TCTCATGGAAATTGGGCTGAGAACACCCAGTTCTAGGATACCCAGGATGATCAAGTGGAGGCAATCAG
AAAATGGCCTATTTCTGGCTCTACGTAGAAACTTAGAAACCTGCCCCCCCCAAATGTCCAGGCTCCAGC
GTTTACTTCCTTACTGTTCTCTCTTTCCCAATAAAGA

c

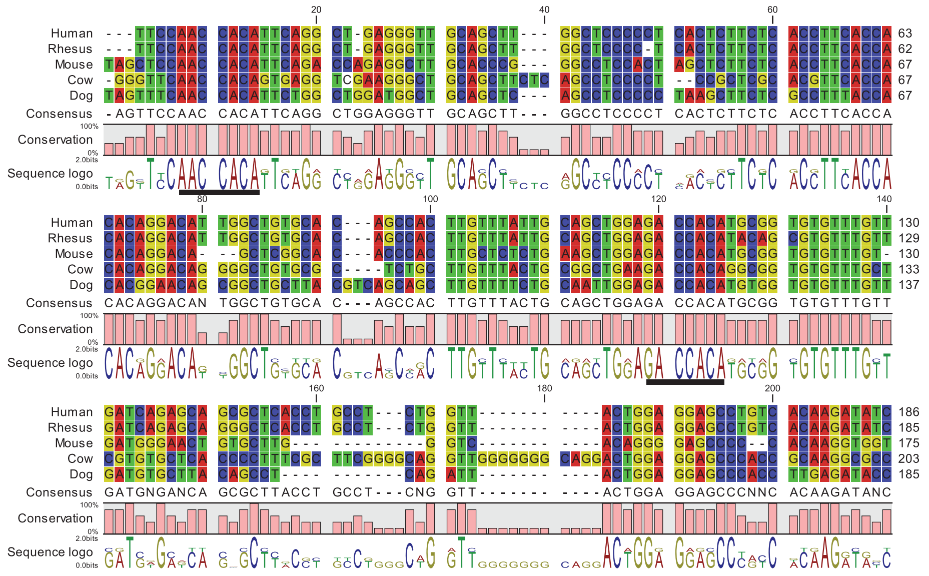

d

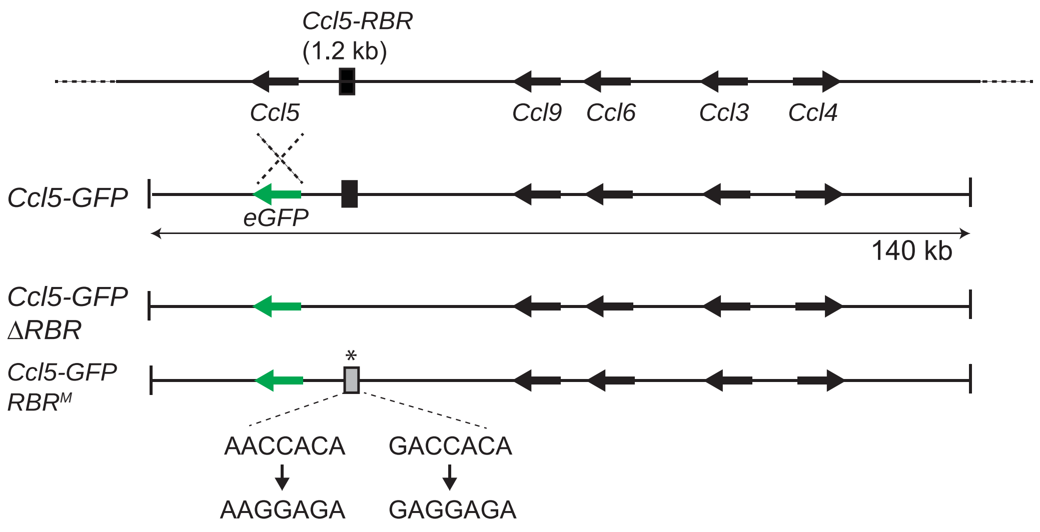

Supplementary Fig. 3.: Ccl5 proximal enhancer (PE) regulates homeostatic CCL5 expression
a Gel images of CBFβ ChIP-qPCR showing CBFβ associations with the CCL5-RBR region in CD4+ and CD8+ T cells.
b Sequences of the 1209 bp Ccl5-RBR region are shown. Sequences with greater conservation are underlined, and Runx Recognition Sequences (RRSs) are shown in bold.
c Alignment of core Ccl5-RBR sequences in the species shown. Two RRSs are underlined.
d Schematic representation of BAC transgenic constructs. The top line shows the locations and transcriptional directions of CC chemokine genes (thick arrows). Black boxes indicate the Ccl5-RBR region. In the Ccl5-GFP BAC transgenic construct, the Ccl5 coding region was replaced with cDNA encoding eGFP (green arrow) on a B6Ng01-015L02 BAC clone spanning 140 kb. In the Ccl5-GFP-ΔRBR transgenic construct, 1.2 kb of the Ccl5-RBR was removed, while two RRSs within Ccl5-RBR have mutated in the Ccl5-GFP-RBRM construct.

a

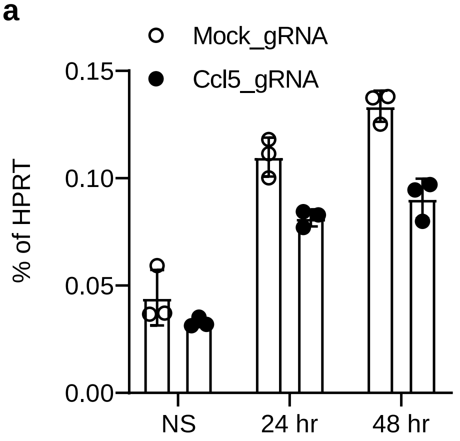

b

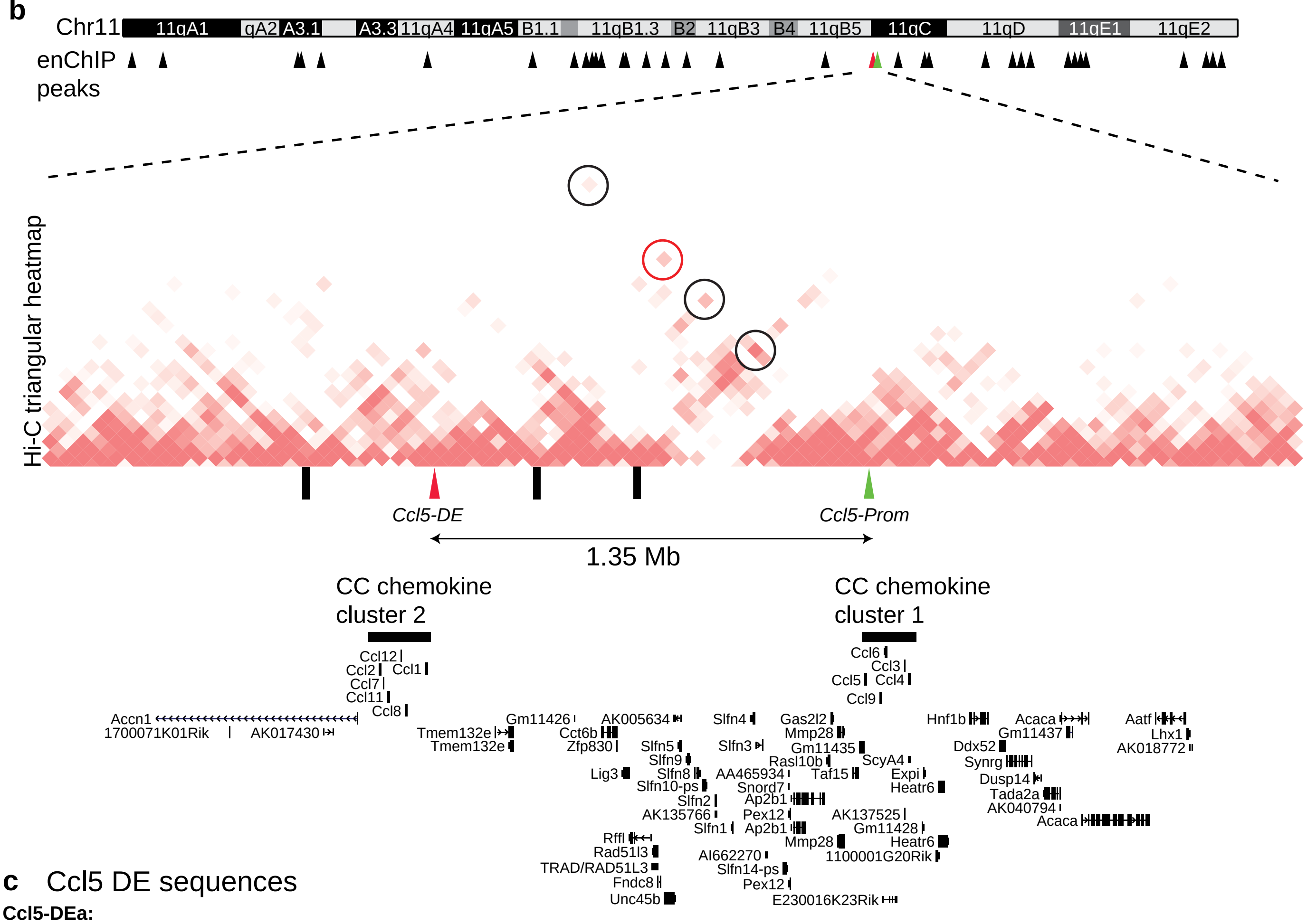

c Ccl5 DE sequences

Ccl5-DEa:

TGGAGTCATCCTAAGATGCTGTCCAGAGGCATTCAGCCCAGCCCTGTCT
ACCACCTGCTACTGAGTTACAGGTCAGGTGGTCCTTCCATAGCAGGCCA
CGGCCAGCCCCTTGGCATGTGTTGAGCATGGCAGAGCTGCAGAGATGA
CTTGATCCATCTCCAGAGTGAAATTCTCTATGTATTCCTTCAGGGAGAGG
AAGAGGAACAGTGACTAGAGCCAGACATAGACACATCAGATCCTAGTTC
TACCGCTTCATCCTGGACAGGTAATACAGGCTTTCTGAGCTGTGTTTCCT
CTTGGACCAAGCACAACTCTCTTTAGTACTGTCAAAATAAACTTCCTAAG
CCTTAGCCTAATTGTGGGTTGTTGGGAGGGCTCCCTGTGATAGGAGCTG
GCTTGAGAGGAAAGGAAAGAATCTCCAGGAGAAGTACTAGGGGGAACC
AAGTGAATCTCAGGGGGACTCCCAGATTATGCAGAGGTAGATCTTGGGG
AGTGGTTTAAATATAGGACGG

Ccl5-DEb:

ATGATGTGTCTTTAACGATGTGGTCACAGTGTCCTTCTGGGTCCTTGATAT
CTGCCCCAGACACAGTTGCCTGAGGTAAGCCTATGTCTATTCACAGTAAA
GATGCCCAGCACCCACA**CACCACA**GAATCCTGAGCCCTGAGCCCTGGC
TTCTTTACTTCCTGTTCC**TGTGGTT**TTTTAGCTGCTTTGTTCTTTTACAGG
AGACTTTCCTGTCTACTGCTTAGTTACAGCACAAAGGACACGGAAGTGTC
TCTCACTACAATAGAAGCAGGCAGAGGTTGTCACAATAGTGACCATCACT
GGTTCTTTCAAGTGCTTCTGAGCCTGAAAGGCCAGCCCGCTGTGCTCAG
GGTGAAAGTACAAATGTCTTGGCCGCACTTACAAAGTGTGAAGTGACGG

d Activated CD8+ T cells (mRNA)

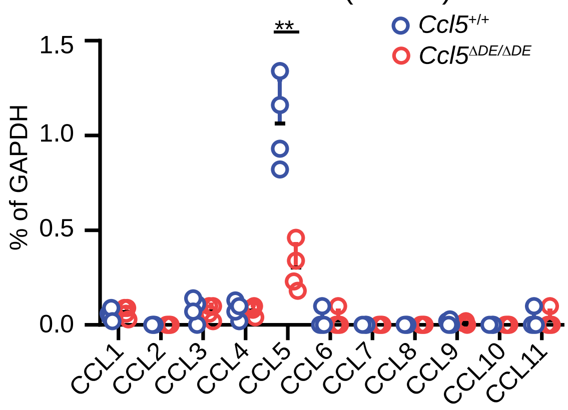

Supplementary Fig. 4.: Identification of Ccl5-distal enhancer (DE) by enChIP
a Summary plot of the amount of Ccl5 mRNA in the 2B4 line after stimulation as assessed by RT-qPCR.
b 34 significant enChIP-seq peaks on entire chromosome 11 that were obtained by enChIP-seq in the 2B4 T cell line are shown as vertical lines on the top line. Positions of the Ccl5 promoter and putative Ccl5-DE are marked by red and green triangles, respectively. Hi-C analysis of mouse Th1 cells using the public database GSE48262 in a genomic area of about 3 Mb encompassing two CC chemokine clusters is shown. Four potential interactions with the Ccl5 promoter (green line) are marked with circles, and the positions of Ccl5 promoter-interacting regions are marked with thick lines. Interactions between Ccl5 promoter and the Ccl5-DE are highlighted in red. Two CC chemokine clusters are marked by thick horizontal lines.
c Sequences of mouse Ccl5-DEa and Ccl5-DEb are shown. Runx Recognition Sequences (RRSs) are shown in bold.
d Relative amounts of mRNA in eleven CC chemokine genes on chromosome 11 in activated CD8+ T cells of Ccl5+/+ and Ccl5ΔDE/ΔDE mice were assessed by RT-qPCR.
Error bars indicate Means ± SD. Statistical significance is measured via unpaired two-tailed Student’s t-tests and is presented as follows: **$p < 0.01$. Source data are provided as a Source Data file.

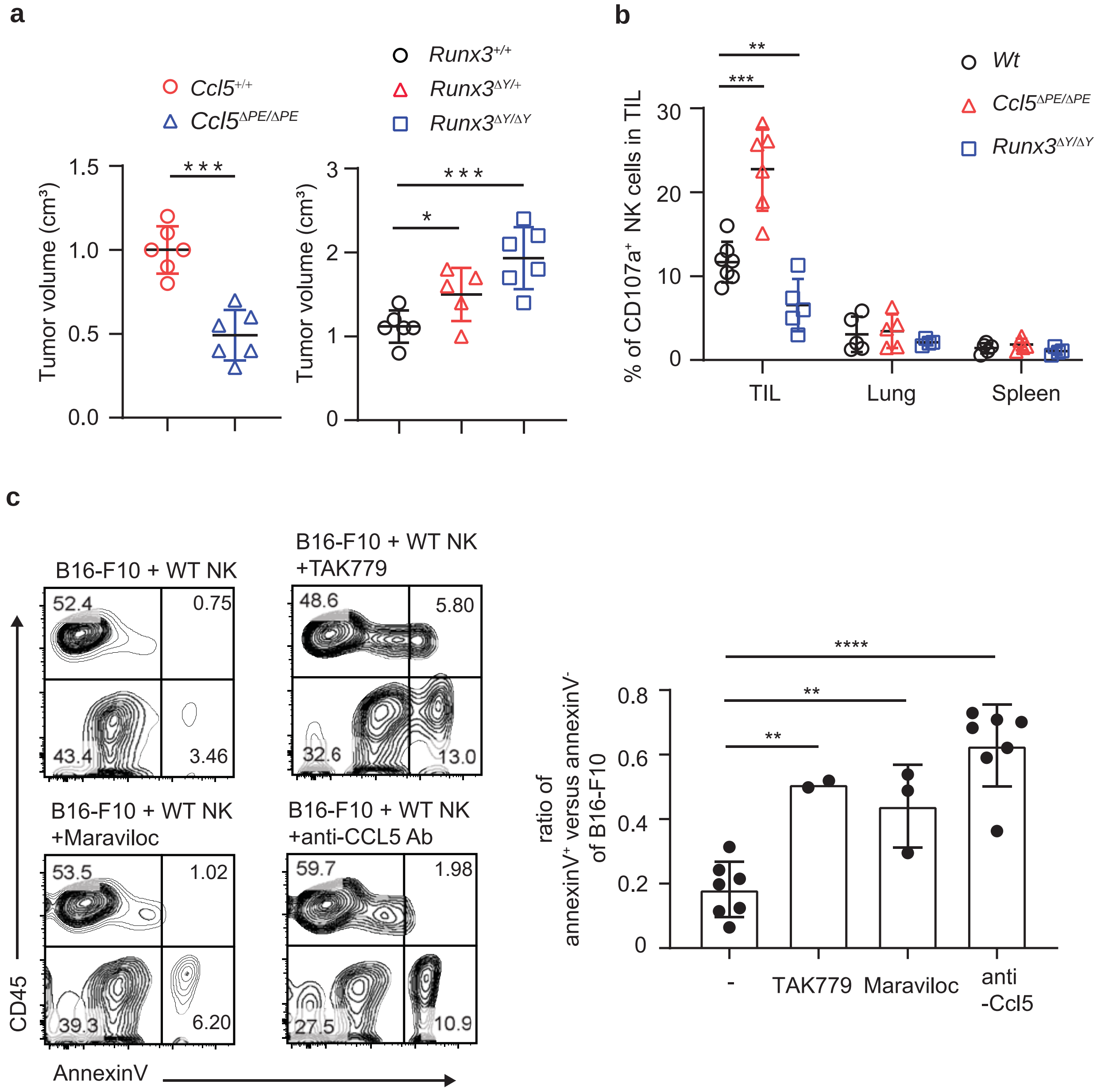

Supplementary Fig. 5.: Functional status changes of NK cells by the CCL5-CCR5 axis
a, b B16-F10 melanoma cells were subcutaneously injected into mice with the indicated genotypes, and tumor-mass diameter (a) and the frequency of CD107a expression in NK cells were measured among tumor infiltrated lymphocytes (TIL), lung NK and spleen NK cells (b).
c Purified NK cells were co-cultured with B16-F10 for four hours before staining with Annexin V to examine the apoptotic B16-F10 frequency as a readout of NK cytotoxicity. NK and B16-F10 cells were defined as CD45+NK1.1+ and CD45−NK1.1− cells, respectively. Maraviroc (a commercial human CCR5 antagonist), TAK779 (a dual inhibitor of human CCR2 and CCR5), and a neutralizing anti-CCL5 antibody were added to inhibit the CCL5-CCR5 interaction. The graph shows a summary of the ratio of Annexin V+ to Annexin V- cells. Numbers in the dot plots indicate the percentage of cells in each quadrant.
a,b,c Error bars indicate Mean ± SD and each dot represents a mouse examined over at least two independent experiments. Statistical significance is measured via unpaired two-tailed Student's t-tests and is presented as follows: $*p < 0.05$, $**p < 0.01$, $***p < 0.001$, $****p < 0.0001$. Source data are provided as a Source Data file.

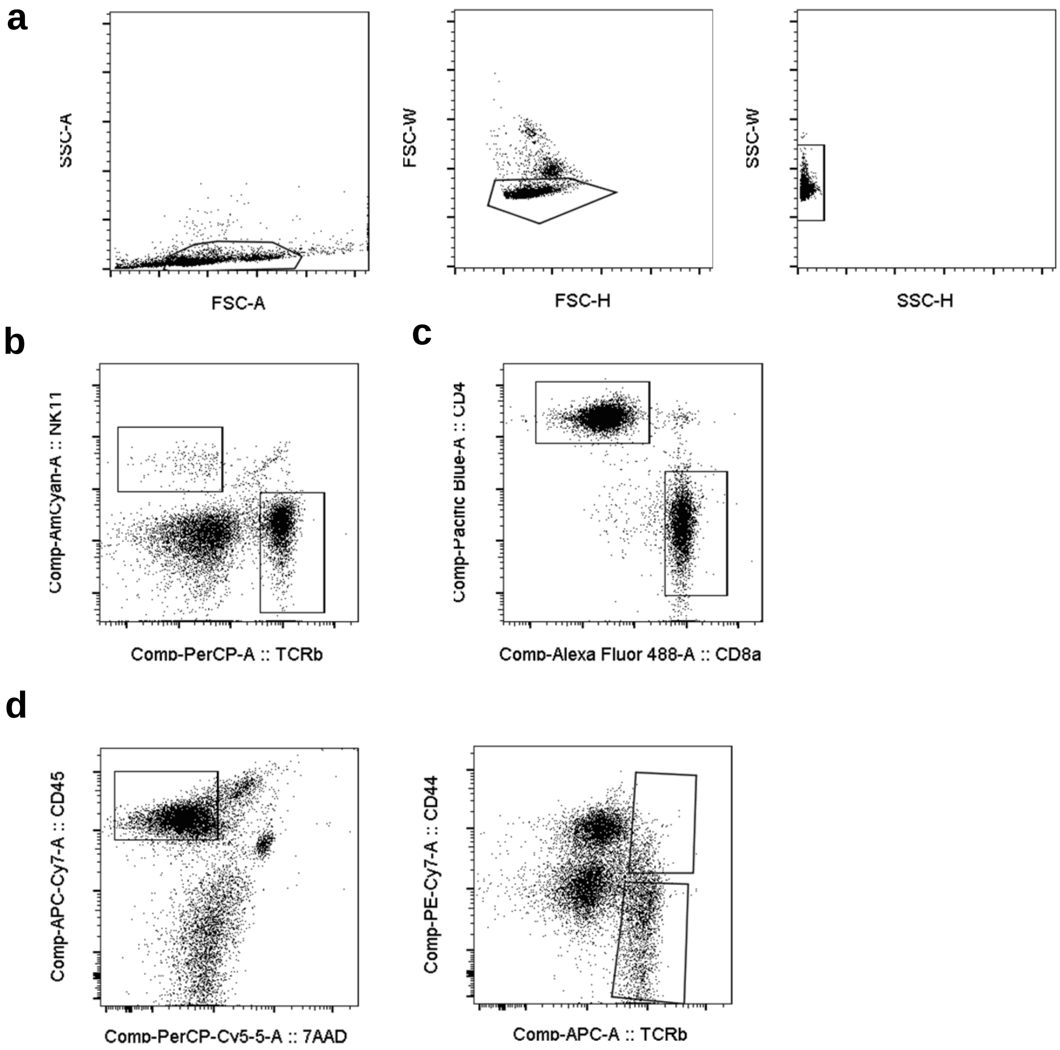

Supplementary Figure 6. Gating strategies used for flow cytometry.
a Gating strategy to splenic and lung.
lymphocyte singlets in all FACS analysis.
b Gating strategies to T cells (NK1.1- TCRb+) presented on Fig. 1.b, 2b,c, 3b,c, 4e, 5d.
c Gating strategy to dissect T cells into CD4+CD8- or CD4-CD8+ populations presented on Fig. 2d, 3d, 4d, 5c, 7b as well as splenic and lung NK cells (NK1.1+ TCRb-) presented on Fig 6c,d,e,f, 7b. The same strategy was used to CD4+ and CD8+ T cells for the *in vitro* assays presented on Sup Fig 1a,b,c, 2a,b, 4c.
d Gating strategy to lung resident memory (Trm) T cells (7AAD- CD45+ TCRb+ CD44+) and presented on Fig 6a,b as well as tumor infiltrating lymphocytes presented on Sup Fig 5b.

Primers used for quantitative qPCR of CC chemokine mRNA (Fig. 5C)

Ccl1-forward	GCCGTGTGGATACAGGATGTTGACAGC
Ccl1-reverse	CAGCTGGGGGATCAGGACAGGAGGAGC
Ccl2-forward	ATGCTTGGCTCAGCAC
Ccl2-reverse	TCAATTTTTGATTTTGAGTGT
Ccl3-forward	CCAAGTCTTCTCAGCGCCATA
Ccl3-reverse	GATGAATTGGCGTGGAATCTTC
Ccl4-forward	TGCTCGTGGCTGCCTTCT
Ccl4-reverse	CTGCCGGGAGGTGTAAGAGA
Ccl5-forward	AGATCTCTGCAGCTGCCCTCA
Ccl5-reverse	GGAGCACTTGCTGCTGGTGTAG
Ccl6-forward	ATGAGAAACTCCAAGACTGCC
Ccl6-reverse	TTATTGGAGGGTTATAGCGACG
Ccl7-forward	AAGATCCCCAAGAGGAATCTCAAG
Ccl7-reverse	CAGACTTCCATGCCCTTCTTTG
Ccl8-forward	TAAGGCTCCAGTCACCTGCT
Ccl8-reverse	TTCCAGCTTTGGCTGTCTCT
Ccl9-forward	ATGAAGCCTTTTCATACTGCCCTC
Ccl9-reverse	TTATTGTTTGTAGGTCCGTGGTTG
Ccl11-forward	CCTGCTGCTTTATCATGACC
Ccl11-reverse	GAGTTTTTGGTCCAGGTGCT
Ccl12-forward	ATTTCCACACTTCTATGCCTCCT
Ccl12-reverse	ATCCAGTATGGTCCTGAAGATCA

Primers used for ChIP-qPCR (Fig. 5D)

Cd4 silencer-forward	CCTTGTGTGGTCCCTCTCTTTG
Cd4 silencer-reverse	GCAACAACCACCCTTCACAGG
Ccl5 PE-forward	GGATCACCTTCTGCTTCCTTACCTCTG
Ccl5 PE-reverse	GCCTAAAATTATCTGTCAACAAACAC
Ccl5 DE-forward	TGTTGAGCATGGCAGAGCTGCAGAGAT
Ccl5 DE-reverse	ATTAGGCTAAGGCTTAGGAAGTTTATTT
Ccl5 promoter-forward	GTTTCCACAAAAGACACCAAAC
Ccl5 promoter-reverse	GAGTGGGACGGCAGATCTGAG
Negative area-forward	CTTGGGATAAGCAGCTCATAGGAGGCC
Negative area-reverse	AGGTTAGCATAGCCAACCCACTGTG

Primers used for 3C-assay qPCR (Fig. 4C)

Ccl5-PE-forward	GGATCACCTTCTGCTTCCTTACCTCTG
Ccl5-PE-reverse	GTGTTTGTTGACAGATAATTTTAGGC
3C-S1	ACTAGGGGGAACCAAGTGAATCTCAG
3C-S2	CTTCCAGAGACCCGCTGTTTAGCAG
3C-S3	GTCACACAGATAGTCACATGAGTAG
3C-AS	GAAAGGATGCTTCATGACTTCACGG

gRNA sequences

Ccl5-PE gRNA1	TGCAGAGGGCCCTACTGACA
Ccl5-PE gRNA2	ACATGCGGTGTGTTTGTGAT
Ccl5-DE gRNA1	ATCTTAGGATGACTCCACCC
Ccl5-DE gRNA1	GGAGTGGTTTAAATATAGGA
Runx3-ΔY gRNA1	TGTGGCGGCCCTACTAAGCA

Supplementary Table 1. Primer sequences for 3C assay related to Fig. 4 C, for a CC chemokine RT-qPCR related to Fig. 5 C, and for ChIP-qPCR related to Fig. 5 E. In addition, a list of gRNA sequences for the generation of knockout mice by Crispr/Cas9.
