# Supplementary material for: Runx-mediated regulation of CCL5 via antagonizing two enhancers influences immune cell function and anti-tumor immunity
Source: Nat Commun. 2020 Mar 26;11:1562. doi: 10.1038/s41467-020-15375-w (PMC7099032; doi:10.1038/s41467-020-15375-w)
Supplement: Supplementary file 3 — Reporting Summary [file 41467_2020_15375_MOESM3_ESM.pdf]

nature research

Corresponding author(s): Wooseok Seo
Last updated by author(s): Mar 3, 2020

Reporting Summary

Nature Research wishes to improve the reproducibility of the work that we publish. This form provides structure for consistency and transparency in reporting. For further information on Nature Research policies, see Authors & Referees and the Editorial Policy Checklist.

Statistics

For all statistical analyses, confirm that the following items are present in the figure legend, table legend, main text, or Methods section.

n/a	Confirmed	
☐	☒	The exact sample size (*n*) for each experimental group/condition, given as a discrete number and unit of measurement
☐	☒	A statement on whether measurements were taken from distinct samples or whether the same sample was measured repeatedly
☐	☒	The statistical test(s) used AND whether they are one- or two-sided<br>*Only common tests should be described solely by name; describe more complex techniques in the Methods section.*
☒	☐	A description of all covariates tested
☒	☐	A description of any assumptions or corrections, such as tests of normality and adjustment for multiple comparisons
☐	☒	A full description of the statistical parameters including central tendency (e.g. means) or other basic estimates (e.g. regression coefficient) AND variation (e.g. standard deviation) or associated estimates of uncertainty (e.g. confidence intervals)
☒	☐	For null hypothesis testing, the test statistic (e.g. *F*, *t*, *r*) with confidence intervals, effect sizes, degrees of freedom and *P* value noted<br>*Give P values as exact values whenever suitable.*
☒	☐	For Bayesian analysis, information on the choice of priors and Markov chain Monte Carlo settings
☒	☐	For hierarchical and complex designs, identification of the appropriate level for tests and full reporting of outcomes
☒	☐	Estimates of effect sizes (e.g. Cohen's *d*, Pearson's *r*), indicating how they were calculated

Our web collection on statistics for biologists contains articles on many of the points above.

Software and code

Policy information about availability of computer code

Data collection	Flow cytometry data was collected by FACS Diva (Version 8).
Data analysis	GraphPad Prism 8 was used for statistical analyses.<br>Flow cytometry data was analyzed by FlowJo (version 10).<br>Hisat2 (version 2.1.0) was used to align reads from RNA-seq and HTSeq (version 0.11.0) to count reads, and further analyzed by EdgeR (3.6.0) for differential expression analysis.<br>bowtie2 (version 2.3.5.1) was used to align reads from DNA-seq and macs2 (version 2.2.5) was used for peak calling.

For manuscripts utilizing custom algorithms or software that are central to the research but not yet described in published literature, software must be made available to editors/reviewers. We strongly encourage code deposition in a community repository (e.g. GitHub). See the Nature Research guidelines for submitting code & software for further information.

Data

Policy information about availability of data

All manuscripts must include a data availability statement. This statement should provide the following information, where applicable:

- Accession codes, unique identifiers, or web links for publicly available datasets
- A list of figures that have associated raw data
- A description of any restrictions on data availability

The data for enChIP-seq, CBFβ ChIP-seq, RNA-seq have been deposited in GEO database under the accession code GSE130600.
https://www.ncbi.nlm.nih.gov/geo/query/acc.cgi?acc=GSE130600
Standard ATAC-seq and histone modifications ChIP-seq data were obtained as fastq files from GSE95237. SATB1 ChIP-seq data were obtained as fastq files from GSE90635. Hi-C analysis of mouse Th1 cells were obtained from GSE48262 and visualized on a web tool (http://promoter.bx.psu.edu/hi-c/).

Field-specific reporting

Please select the one below that is the best fit for your research. If you are not sure, read the appropriate sections before making your selection.

☒ Life sciences ☐ Behavioural & social sciences ☐ Ecological, evolutionary & environmental sciences

For a reference copy of the document with all sections, see nature.com/documents/nr-reporting-summary-flat.pdf

Life sciences study design

All studies must disclose on these points even when the disclosure is negative.

Sample size	Sample size for each experiment is indicated in the figures and figure legends for each experiment. No statistical methods were used to predetermine sample size.
Data exclusions	No data was excluded in this manuscript.
Replication	All experiments described in this manuscript have been performed more than twice. All attempst at replication were successful.
Randomization	No systemic randomization was used for animal studies since methods such as pathological scoring including subjective measures were not used in this study. However, standard procedures to remove experimental bias were in order such as mouse sexes, ages, litters and cages. No randomization was used for experiments other than animal studies.
Blinding	No blinding test was done in this study since methods such as pathological scoring including subjective measures were not used in this study.

Reporting for specific materials, systems and methods

We require information from authors about some types of materials, experimental systems and methods used in many studies. Here, indicate whether each material, system or method listed is relevant to your study. If you are not sure if a list item applies to your research, read the appropriate section before selecting a response.

Materials & experimental systems

n/a	Involved in the study
☐	☒ Antibodies
☐	☒ Eukaryotic cell lines
☒	☐ Palaeontology
☐	☒ Animals and other organisms
☒	☐ Human research participants
☒	☐ Clinical data

Methods

n/a	Involved in the study
☐	☒ ChIP-seq
☐	☒ Flow cytometry
☒	☐ MRI-based neuroimaging

Antibodies

Antibodies used

For in vitro T cell culture, the following antibodies were used: 2 µg/ml anti-CD3e antibody (553058, BD Biosciences) with 2 µg/ml soluble anti-CD28 antibody (553295, BD Biosciences).

For flow cytometry analyses, antibodies were purchased from BD Bioscience, Biolegend, ThermoFisher : Annexin V (Biolegend), CCL1 (148107), CCL5 (2E9/CCL5), CD4 (RM4-5), CD8a (53-6.7), CD8β (YTS156.7.7), CD11b (M1/70), CD25 (PC61), CD44 (IM7), CD45.2 (104), CD69 (H1.2F3), CD103 (M290), IFNγ (XMG1.2), NK1.1 (PK136), Gr-1 (RB6-8C5), Ter119 (Ter119), TCRβ (H57-597), TCRγδ (GL3) and CD107a (eBioH4A3) which were used at 0.5 µg/mL.

For ChIP assays, the following antibodies were purchased: anti-FLAG (M2) from Sigma, anti-SATB1 (ab70004) from Abcam and anti-CBFb (in house).

Validation

Hamster anti-mouse CD3e antibody (553058, BD Biosciences)
The testing is noted in the following website:https://www.bdbiosciences.com/us/applications/research/t-cell-immunology/th-1-cells/surface-markers/mouse/purified-hamster-anti-mouse-cd3e-145-2c11/p/553058

Hamster anti-mouse CD28 antibody (553295, BD Biosciences)
The testing is noted in the following website:https://www.bdbiosciences.com/us/applications/research/t-cell-immunology/regulatory-t-cells/surface-markers/mouse/purified-hamster-anti-mouse-cd28-3751/p/553295

Annexin V (640937, Biolegend)
The testing is noted in the following website:https://www.biolegend.com/en-us/products/brilliant-violet-510-annexin-v-9791

Goat anti-mouse CCL1 (PA5-47952)

The testing is noted in the following website:https://www.thermofisher.com/antibody/product/CCL1-Antibody-Polyclonal/PA5-47952

Mouse anti-mouse CCL5 (2E9/CCL5)
The testing is noted in the following website:https://www.biolegend.com/en-us/products/pe-anti-mouse-ccl5-rantes-antibody-10420

Rat anti-mouse CD4 (RM4-5)
The testing is noted in the following website:https://www.biolegend.com/en-us/products/brilliant-violet-421-anti-mouse-cd4-antibody-7349

Rat anti-mouse CD8a (53-6.7)
The testing is noted in the following website:https://www.bdbiosciences.com/us/reagents/research/antibodies-buffers/immunology-reagents/anti-mouse-antibodies/cell-surface-antigens/pe-rat-anti-mouse-cd8a-53-67/p/561095

Rat anti-mouse CD8b (YTS156.7.7)
The testing is noted in the following website:https://www.biolegend.com/en-us/products/pe-anti-mouse-cd8b-antibody-4476

Rat anti-mouse CD11b (M1/70)
The testing is noted in the following website:https://www.biolegend.com/en-us/products/apc-anti-mouse-human-cd11b-antibody-345

Rat anti-mouse CD25 (PC61)
The testing is noted in the following website:https://www.biolegend.com/en-us/products/fitc-anti-mouse-cd25-antibody-422

Rat anti-mouse CD44 (IM7)
The testing is noted in the following website:https://www.biolegend.com/en-us/products/apc-anti-mouse-human-cd44-antibody-312

Mouse anti-mouse CD45.2 (104)
The testing is noted in the following website:https://www.biolegend.com/en-us/products/pacific-blue-anti-mouse-cd45-2-antibody-3108

Hamster anti-mouse CD69 (H1.2F3)
The testing is noted in the following website:https://www.bdbiosciences.com/us/applications/research/t-cell-immunology/regulatory-t-cells/surface-markers/mouse/pe-cy7-hamster-anti-mouse-cd69-h12f3/p/552879

Rat anti-mouse CD103 (M290)
The testing is noted in the following website:https://www.bdbiosciences.com/us/applications/research/t-cell-immunology/regulatory-t-cells/surface-markers/mouse/apc-rat-anti-mouse-cd103-m290/p/562772

IRat anti-mouse FNγ (XMG1.2)
The testing is noted in the following website:https://www.biolegend.com/en-us/products/fitc-anti-mouse-ifn-gamma-antibody-995

Mouse anti-mouse NK1.1 (PK136)
The testing is noted in the following website:https://www.biolegend.com/en-us/products/pe-anti-mouse-nk-1-1-antibody-431

Rat anti-mouse Gr-1 (RB6-8C5)
The testing is noted in the following website:https://www.biolegend.com/en-us/products/apccyanine7-anti-mouse-ly-6gly-6c-gr-1-antibody-3935

Rat anti-mouse Ter119 (Ter119)
The testing is noted in the following website:https://www.biolegend.com/en-us/products/apc-anti-mouse-ter-119-erythroid-cells-antibody-1863

Hamster anti-mouse TCRβ (H57-597)
The testing is noted in the following website:https://www.bdbiosciences.com/us/reagents/research/antibodies-buffers/immunology-reagents/anti-mouse-antibodies/cell-surface-antigens/fitc-hamster-anti-mouse-tcr-chain-h57-597/p/553171

Hamster anti-mouse TCRγδ(GL3)
The testing is noted in the following website:https://www.biolegend.com/en-us/products/pe-anti-mouse-tcr-gamma-delta-antibody-2421

Rat anti-mouse CD107a (eBioH4A3)
The testing is noted in the following website:https://www.bdbiosciences.com/us/reagents/research/antibodies-buffers/immunology-reagents/anti-mouse-antibodies/cell-surface-antigens/bv421-rat-anti-mouse-cd107a-1d4b/p/564347

Mouse anti-mouse anti-FLAG (M2, Sigma)
The testing is noted in the following website:https://www.sigmaaldrich.com/catalog/product/sigma/f3165?lang=ja&region=JP

Rabbit anti-mouse anti-SATB1 (ab70004, Abcam)
The testing is noted in the following website:https://www.abcam.com/satb1-antibody-ab70004.html

Eukaryotic cell lines

Policy information about cell lines

Cell line source(s)	2B4 mouse T cell line was kindly provided by Dr. Takashi Saito (RIKEN), YAC-1 cell line was kindly provided by Dr. Shin-ichiro Fujii (RIKEN) and B16-F10 mouse melanoma was purchased from RIKEN BRC.
Authentication	None of the cell lines used have been authenticated.
Mycoplasma contamination	Cell lines were not tested for Mycoplasma contamination.
Commonly misidentified lines (See ICLAC register)	No commonly misidentified cell lines were used.

Animals and other organisms

Policy information about studies involving animals; ARRIVE guidelines recommended for reporting animal research

Laboratory animals	Mice used in this study include wild type, Runx1ΔV, Runx3ΔV, CbfβF/F:Cd4-cre and Satb1F/F:Cd4-cre, Ccl5ΔPE/ΔPE and Ccl5ΔDE/ΔDE. Both male and female sexes were used at between 4 weeks to 4 months old. Mice were maintained in the animal SPF facility at the RIKEN IMS, which provides housing conditions at a constant temperature (24oC ± 2oC), relative humidity (55 ±10%) and ventilation rate (7 air changes/hr).
Wild animals	No wild animals were used in this study.
Field-collected samples	No field collected samples were used in this study.
Ethics oversight	All animal procedures were in accordance with protocol approved by the institutional Animal Care and Use Committee (IACUC) of RIKEN Yokohama Branch.

Note that full information on the approval of the study protocol must also be provided in the manuscript.

ChIP-seq

Data deposition

☒ Confirm that both raw and final processed data have been deposited in a public database such as GEO.

☒ Confirm that you have deposited or provided access to graph files (e.g. BED files) for the called peaks.

Data access links<br>*May remain private before publication.*	GSE130600
Files in database submission	GSM3734262 mock gRNA<br>GSM3734263 Ccl5 promoter gRNA<br>GSM3744544 CBFb CD4+ T input<br>GSM3744545 CBFb CD4+ T ip<br>GSM3744546 CBFb CD8+ T input<br>GSM3744547 CBFb CD8+ T ip<br>GSM3938822 Lung_WT1<br>GSM3938823 Lung_WT2<br>GSM3938824 Lung_KO1<br>GSM3938825 Lung_KO2<br>GSM3938826 Spleen_WT1<br>GSM3938827 Spleen_WT2<br>GSM3938828 Spleen_KO1<br>GSM3938829 Spleen_KO2
Genome browser session (e.g. UCSC)	No longer applicable

Methodology

Replicates	ChIP-seq experiments were performed twice with different batches and one representative result were deposited.
Sequencing depth	Libraries were sequenced to generate single-end 50bp and sequencing depth for each ChIP-seq sample was between 15-20 millions reads.
Antibodies	anti-FLAG (M2) from Sigma and anti-CBFb (in house).
Peak calling parameters	ChIP peaks were identified using MACS2 with the default parameters

Data quality | Peaks were called using MACS2 (version 2.1.1) with the upper (50-fold) and lower (2-fold) limit for model building.

Software | Hisat2 was used to align read to the mouse genome (mm10). ChIP -seq peaks were identified using MACS2.

Flow Cytometry

Plots

Confirm that:

☒ The axis labels state the marker and fluorochrome used (e.g. CD4-FITC).

☒ The axis scales are clearly visible. Include numbers along axes only for bottom left plot of group (a 'group' is an analysis of identical markers).

☒ All plots are contour plots with outliers or pseudocolor plots.

☒ A numerical value for number of cells or percentage (with statistics) is provided.

Methodology

Sample preparation	Cell suspension from spleen and lung were prepared by mashing tissues through a 70 µm cell strainer. Red blood cells were removed before analysis.
Instrument	Data collection was performed by FACS-Canto II or FACS-Aria (BD Biosciences).
Software	To collect FACS data, we used FACS Diva software. Data were analyzed by FlowJo software.
Cell population abundance	Purities of post-sort samples were checked by FACS analysis to ensure more than 99% of the purity.
Gating strategy	FSC/SSC were used for lymphocytes, FSC-H/FSC-W for single cells and 7AAD for viable cells.

☒ Tick this box to confirm that a figure exemplifying the gating strategy is provided in the Supplementary Information.
