# Supplementary material for: Runx-mediated regulation of CCL5 via antagonizing two enhancers influences immune cell function and anti-tumor immunity
Source: Nat Commun. 2020 Mar 26;11:1562. doi: 10.1038/s41467-020-15375-w (PMC7099032; doi:10.1038/s41467-020-15375-w)
Supplement: Supplementary file 2 — Peer Review File [file 41467_2020_15375_MOESM2_ESM.pdf]

Reviewers' comments:

Reviewer #1 (ATAC-seq, transcriptome analysis, RUNX) (Remarks to the Author):

The manuscript entitled “Suppression of CCL5 by RUNX via antagonizing two enhancers is crucial to enhance tumor immunity” by Seo et al, delineates expression regulation of the cytokine Ccl5 in mouse T and NK cells. The authors identified two enhancer elements that control Ccl5 expression in a stage- and cell type-specific manner. They further demonstrate the importance of the proximal enhancer (Ccl5-PE) for Ccl5 expression under steady state (homeostatic) conditions and the distal enhancer, located 1.35 Mb away (Ccl5-DE), for Ccl5 regulation in activated T cells. They use ATAC-seq and histone marked chromatin Chip-seq methods to characterize these enhancers and 3C long-range looping assay to demonstrate looping of Ccl5-DE with the Ccl5 promoter in a Satb1-dependent manner. Finally, they demonstrate the importance of these Ccl5 enhancers in regulation of antitumor activity of tumor-infiltrating NK cells in mouse models of melanoma skin tumor formation and lung metastases. The manuscript is interesting, encompasses a large amount of work, using up-to-date biochemical and biological methods. The manuscript has potential implications regarding the impact of Ccl5 expression in various immune cells on their role in immunity, inflammation and cancer. Nevertheless, there are certain points that need to be corrected and clarified as detailed below.
Major points:

1. Page 6 lines 124-127: While CBFβ binding (Fig. 2A) is shown for both CD4+ and CD8+ T cells, the pattern of ATAC tracks are shown only for CD8+ T cells (naïve, effector and memory). Also, the pattern of H3K4me1 and H3K27Ac tracks is shown only for CD8+ memory T cells. To obtain a complete picture, the authors should also display the tracks of ATAC, H3K4me1 and H3K27Ac in naïve and memory CD4+ T cells and these histone marks in naïve CD8+ T cells. Furthermore, the authors state “the analysis of ATAC-seq database showed that the Ccl5-RBR becomes more accessible in effector/memory T cells than in naïve T cells”, but the actual difference does not look impressive. The authors should give an estimate of the extent to which it becomes “more accessible”.
2. Page 6 line 128: The Ccl5-RBR actually contains four RRS, not just the two marked in Fig. S3B and S3C and schematically illustrated in Fig. 3A. An additional ACCACA is found 50 bp downstream of the first ACCACA that is marked in bold letters and another RRS, TGCGGT, is adjacent to the second marked ACCACA.
3. Page 6 line 129: The authors mention rs11652536, a SNP located in CCL5-RBR, that is associated with the severity of liver fibrosis in humans. The relevance of this statement to the data in this manuscript is unclear and could be misleading, since it is completely unknown whether this SNP affects CCL5 expression in human T or NK cells that are the subject of the present work and whether the liver fibrosis is mediated by these immune cells. Also, this SNP was not reported in a genome-wide associated study.
4. Page 6 line 140: In Fig. 2C, the heading should be changed from “Activated T cells” to “Activated CD8 T cells”.
5. Page 6 line 153: The section is written in a convoluted way. To clarify the authors should instead of “Spleen” in Figures 2B and 3B, show data in CD4+ and CD8+ T cells, as they did in Figure 1.
6. Page 6, line 162: It is unclear whether the cells referred to as “CD44+ memory-like T cells” include CD4+CD44+, CD8+CD44+ or both cell types. This point is important as expression of Ccl5 in activated CD4+ T cells differs considerably from that in activated CD8+ T cells (Fig. 3D). Presumably, the spleen cells depicted as “TCRβ+” in the 2 right panels with dot plots of Fig. 3B are CD8+CD44+, if so they should be mark “TCRβ+CD8+. The authors should specify in all places where CD44+ cells are mentioned, whether they refer to CD4, CD8 or to both types of memory-like T cells.
7. Page 8 line 208: It is unclear from the 3C assay how Ccl5 expression was increased in CbfF/F;Cd4-Cre in activated CD4+ T cells (Fig. 3D), while there were merely a minor signals in the 3C assay between the Ccl5 promoter and the Ccl5-DE in these cells (Fig. 4C). The authors should clarify this

ambiguity.
8. Page 9 line 218-219: In activated CD8+ T cells; DEb element has a clear repressive activity on Ccl5 expression as depicted in Fig. 4D. But besides showing CBFb binding to this element (ChIP-seq Fig. 4A), no data is given as to the presence of RRS in this region, nor for the effect of introducing a DEb-specific mutation in such an RRS. It is thus unclear whether DEb repressive activity depends on CBFb binding to this element. Consequently, the conclusions in the Abstract (lines 22-23) and Introduction (page 4 lines 69-70) that “both enhancers are negatively regulated by RUNX/CBFβ complexes” have not been established experimentally and the DEb element function is not characterized. These flaws should be amended.
9. Page 9 lines 222-238: The authors conclude that the looping of the Ccl5 promoter with Ccl5-DE is dependent on Satb1 which presumably associates with Cbfb at the Ccl5-DE. They show that this Satb1-mediated looping is responsible for the enhanced expression of Ccl5 in Satb1F/F;Cd4-Cre CD8+ T cells. What is not demonstrated and critically missing is an experiment showing that the same holds for CbfF/F;Cd4-Cre CD4+ and/or CD8+ T cells. It is particularly important because Ccl5-DE does not harbor a RRS, as stated in the Discussion. The reader would also be interested to know whether the Ccl5 promoter bears a RRS.
10. Page 9 line 234 (related to Fig. 5D): In the Figure itself Ccl5 expressing cells in Satb1F/F; CD4-Cre CD44+ CD8+ T cells appears to be increased ~5-fold, yet in the text it is written “Homeostatic Ccl5 expression in CD44+ memory CD8+ T cells was unaffected, or tended to increase in Satb1-deficiency”. This discrepancy should be dealt with. Also, Fig. 4D does not include a histogram to show the statistics of the data depicted as a dot plot.
11. Page 10 lines 263-265: The finding that in lung NK “about two thirds of total mRNAs either increased or decreased by the absence of Ccl5-PE” is quite surprising and very unusual. The authors should analyze whether this applies also to mRNAs of the three Runx genes Runx1, Runx2 and Runx3 and whether they are increased or decreased? It is also, important to demonstrate haw expression of these genes is affected in Ccl5-PE-deficient activated CD4+ and CD8+ T cells? This is particularly important for Runx3 known to regulates proliferation of activated NK, CD4+ Th1 and CD8+ T cells.
12. Page 12 lines 312-314: The authors should demonstrate whether the conclusion about the impact of blocking Ccl5 expression in lung NK-mediated killing of melanoma cells also apply to CD8+ T cells.
13. Page 12 lines 321-329: It is entirely unclear how the authors envision the mechanism undelaying enhancers-mediated regulation of Ccl5 expression by Satb1 and Cbfb in a situation where the Ccl5-DE lacks an RRS, but Cbfb is required for repressing Ccl5 expression in NK and T cells.
In this context, the statement in lines 327-328 “The lack of RUNX-recognition sites in the Ccl5-DE indicates that the ways by which RUNX-CBFb complexes are recruited to the two Ccl5 enhancers presumably differ” is not sustained and thus vague.
14. Finally, while the title of the manuscript implies an important role for RUNX in suppressing Ccl5 expression, in the discussion (page 13 line 330) it is stated “It is unclear how RUNX-mediated suppression is cancelled in Ccl5 expressing cells”. The authors should consider rephrasing their conclusions and title to reflect the finding described in the manuscript.

Minor:

1. Page 4 line 79: Add the word “factor” after “transcription”.
2. Page 5 line 109: Remove the word “of” before “in suppressing CC chemokines”.
3. Page 6, line 155: the word “CD8” after “activated” should be added.
4. Fig. 3B right panel histogram: The red triangles (CbfbF/F; CD4-Cre) and the blue squares (Ccl5ΔPE/ΔPE) should be switched.

Reviewer #2 (Cancer immunity, NK, transcription regulation) (Remarks to the Author):

The authors investigate the regulation of CCL5 by naïve (homeostatic expression) vs activated (induced expression) T cells and the role of Runx proteins in this context. They also study how homeostatic CCL5 expression impact on anti-tumor immunity in the context of B16 melanoma.

The authors claim the identification of two enhancers: one that is involved in the regulation of the homeostatic expression of CCL5 and termed CCL5-RBR or CCL5-PE, and one that is involved in the regulation of the induced expression of CCL5, termed CCL5-DE.

The MS provides an interesting hypothesis, but the data provided need to be substantiated and clarified to better support it.

General comments
In this work, the authors produce a large series of 6 novel mouse mutants, including BAC transgenic mutants and gene targeted mice using the Crispr/Cas9 technology. This a huge effort. However, the authors do not provide any characterization of the impact of the gene modifications on the immune system in those mice so that the usefulness of the gene targeted mice is limited (more details below).

The accessibility of data would gain substantially, if the authors would unify the presentation of the data. In figure 1 the expression of CCL5 is shown for CD4+CD8-TCRb+ and CD8+TCRb+ T cells (Figure 1b middle histograms). The analysis of the latter cells is more detailed: expression of CCL5 in relation to expression of CD44 (Figure 1b, right dotplots).
In subsequent figures, the representation changed as CCL5 or CCL5-GFP expression by all TCRb+ cells is shown in relation to CD44: Figure 2B and C, Figure 3 B, Figure 5D, FS1C. It is not explained why this is so.
The authors need to state if the observed effects are similar (or not!) in CD4 as well as CD8 T cells and provide those data on CD4 and CD8 T cells, separately, in a supplemental figure.
Moreover, when the authors analyze activated T cells, they separate CD4 from CD8 T cells.

! It is not always clearly stated how many independent experiments have been performed. Please, rectify, where the information is missing.

Specific comments/The data will be reviewed figure by figure:

Figure 2
Why do the authors use different mouse mutants when analyzing the homeostatic expression of CCL5 (CCL5-GFP-ΔRBR) vs induced CCL5 expression (CCL5-GFPRBRM)?
In naïve mice, CCL5-GFP expression is abolished in absence of the identified enhancer (CCL5-GFP-ΔRBR), but how is it when the enhancer is present, but only the two binding sites for the Runx repressor (RRS) are mutated?
One would expect an increased CCL5-GFP expression - actually, as seen in the activated T cells from these mice (CCL5-GFPRBRM). The analysis of naïve CD8 T cells from CCL5-GFPRBRM would also help interpret the data derived from activated T cells.

How do the authors explain that CCL5-GFP is not expressed in activated T cells (- absence of CCL5-DE), while in absence of RUNX function (Cbfb mutation) it is induced – in an apparent absence of other Runx-ChIP peaks with RRS and open in effector/memory cells ?

Figure 3

Generation of CCL5ΔPE/ΔPE mice = mice carrying a deletion that removes both RSS sites from the RBR region in the identified CCL5 enhancer.

CCL5ΔPE/ΔPE mice show a similar phenotype than CCL5-GFP-ΔRBR (and naïve CCL5-GFPRBRM also?) mice. Again, how do the authors explain that CCL5 is expressed in naïve T cells from CCL5ΔPE/ΔPE mice when Cbfb is deleted – i.e., the repressor and its binding site are missing?
The last phrase on page 7 (line 173 onwards) is not clear.

B: In the diagram on the right, the red triangles and blue squares are mixed up.
Also, please, unify the color code in this figure aside from the mix-up. This would enhance readability of the data.

C: On the right "total" splenocytes are shown where it should be TCRb- splenocytes in order to restrict the analysis on NK cells and to remove NK-T cells.

The legend of the y-axes on both diagrams needs to be corrected and completed.

Figure 5

C: A negative control is missing to be able to interpret the data shown: do all CD8+ T cells from Sat1b1f/f;Cd4-cre mutant mice express IFNgamma upon stimulation but at a lower level than WT T cells or do only 60% of the activated mutant CD8+T cells express it?

D: CD8+ T cells from Sat1b1f/f;Cd4-cre mutant mice seem to harbor an increased CD44+ population. Are these cells pre-activated? Please, provide data on their activation status compared to WT cells.
Please, provide statistics of the % of CCL5+ cells among naïve CD44+CD8+ T cells.
What means "multiple independent experiments" in the figure legend? Give numbers.

Figure 6

A: Surprisingly, in the controls Trm T cells seem to lack CD69 and CD103 expression. However, the majority of Trm T cells in the lung express these markers (review Mueller and Mackay, 2016).
Please, comment.

Also, the gates in the dotplot on the right are wrongly placed: they cut the populations in half. This needs to be rectified. Adjust the statistics accordingly

D: The aim is to assess the functional status of the NK cells from a novel mutant mouse line. It is surprising that the authors do not characterize the NK cells in these mice, first: their differentiation status and repertoire expression, developmental stages in the bone marrow and tr-NK cells in the liver. This needs to be done.

RNA-seq-based analyses allow to reveal functional potential of NK cells. The data obtained are interesting, but only suggestive as RNA expression does not necessary translates into changes on the protein level or functional consequences.
In order to assess the true functional status of mutant vs control NK cells it is necessary to perform in vitro assays with purified NK cells.

E: Why do the authors say that the difference of NK cells between WT and CCL5-PE KO mice is as much distinct as the NK cells between spleen and lung (page 11, lines 268/269)?
First, the data do not support that, and second, why would that be of any interest?

The phrase “These findings…potent activities of NK cells” (lines 271-274) is wrong. It is neither clear at this point, if there are effectively differences, nor if they would be NK cell intrinsic or due to the aberrant activities of accessory cells.

Figure 7
Line283: “Given altered properties…” this phrase is wrong! This has not been shown, only suggestive data presented (see comments Figure 6E). Add the acquired data or modify the phrase.

The lack of a characterization of the three novel mutant mouse lines (CCL5ΔPE/ΔPE, CCL5ΔDE/ΔDE, Runx3ΔY/ΔY) does not allow to asses fully the role of CCL5 in these assays. Are only NK cells affected or also T cells (esp CD8+ T cells) or myeloid cells? Are those cells spontaneously activated? Secreting cytokines?

It seems clear that CCL5ΔPE/ΔPE mice develop fewer and Runx3ΔY/ΔY mice more B16 lung metastases than controls, but it remains unclear why. Both molecules exert activities on several cell types.
A clear support for the last statement (lines 312-314) is missing.

Besides providing a general characterization of the mouse mutants, it is necessary to provide more data on TIL-NK cells (absolute numbers, phenotype) and other TIL in the skin B16 experiments (Figure S5), especially TIL-CD8+ T cells (numbers and their activation status - IFNgamma expression levels, cytotoxicity).

FS5C: why didn’t the author use NK cells from CCL5ΔPE/ΔPEand CCL5ΔDE/ΔDE mice to assess the role of CCL5 here?
What was the purity of NK cells used?

The diagram on the right is not clear: we are looking on the ratio of annexin V+ vs annexin V- of what cells – B16 or NK cells or both? Better calculate the % of Annexin V+ B16 cells.

Minor:
Typo line 109: remove “of”
Line 285: what means “physiological” in this context?

Reviewer #3 (Tumor immunity, CCL5) (Remarks to the Author):

The paper of Seo, Taniguchi and colleagues reports on the novel role of RUNX/CBFb interactions in regulating host’s CCL5 production and tumor growth in lung metastasis model of B16-F10. The current manuscript contains multiple interesting elements, which could help to explain current controversies regarding the roles of CCL5 and CCR5 in cancer immunity and cancer progression. Unfortunately, in its current form, the paper does not provide a clear picture and offers only very limited connection between different reported findings. Addressing the following issues could enhance the appeal and predicted impact of the paper:
1) The narrative of the paper (including the abstract) is dense and does not present a clear story. A significant amount of editing could help to highlight the key elements of the paper and connect its different element.
2) The key proposal (title) that the modulation of CCL5 by RUNX/CBFb is responsible for the observed differences in B16-F10 growth is not fully supported by data, and seems to be based on the observed correlations rather than mechanistic evidence. Experiments showing that the RUNX status regulates

the B16 growth rate only in the absence but not in the presence of CCL5 (or CCR5) blockade, would help to support the central message. Development of double knockout mice deficient in both RUNX and CCL5 could provide even stronger evidence.

3) Taking into account that the paper addresses the role of host-derived CCL5, it is unclear why only a single model (i.v. B16-F10) is used to support the central message of the paper and evaluate the impact on tumor growth.

4) The paper implicates that the impact of RUNX is mediated by altered immune status is based on the observed associations, but lacks comparison of immunodeficient and immunocompetent animals.

5) The introduction of the paper very dense and describes multiple, apparently contrasting earlier reports on the role of CCL5 in antitumor immunity, but, in its current form, it does not provide the reader with a hypothesis leading the authors to the proposed studies. Similarly, the discussion could also be enhanced to provide the reader with better understanding of how the current findings help to interpret the previously reported data.

6) I suggest to discuss the current observations in the context of the seminar report from Jurgen Becker showing that the presence of the CCR5Delta32 polymorphism in patients with stage IV melanoma results in a selectively decreased survival following immunotherapy , although it does not affect the melanoma survival in untreated patients (Cancer Immunol Immunother. 2008 May;57(5):685-91.)

Reviewer #1 (ATAC-seq, transcriptome analysis, RUNX) (Remarks to the Author):

The manuscript entitled “Suppression of CCL5 by RUNX via antagonizing two enhancers is crucial to enhance tumor immunity” by Seo et al, delineates expression regulation of the cytokine Ccl5 in mouse T and NK cells. The authors identified two enhancer elements that control Ccl5 expression in a stage- and cell type-specific manner. They further demonstrate the importance of the proximal enhancer (Ccl5-PE) for Ccl5 expression under steady state (homeostatic) conditions and the distal enhancer, located 1.35 Mb away (Ccl5-DE), for Ccl5 regulation in activated T cells. They use ATAC-seq and histone marked chromatin Chip-seq methods to characterize these enhancers and 3C long-range looping assay to demonstrate looping of Ccl5-DE with the Ccl5 promoter in a Satb1-dependent manner. Finally, they demonstrate the importance of these Ccl5 enhancers in regulation of antitumor activity of tumor-infiltrating NK cells in mouse models of melanoma skin tumor formation and lung metastases.

The manuscript is interesting, encompasses a large amount of work, using up-to-date biochemical and biological methods. The manuscript has potential implications regarding the impact of Ccl5 expression in various immune cells on their role in immunity, inflammation and cancer.

We wish to express our appreciation to the reviewer for his/her insightful comments on our paper.

Nevertheless, there are certain points that need to be corrected and clarified as detailed below.

Major points:

1. Page 6 lines 124-127: While CBFb binding (Fig. 2A) is shown for both CD4+ and CD8+ T cells, the pattern of ATAC tracks are shown only for CD8+ T cells (naïve, effector and memory). Also, the pattern of H3K4me1 and H3K27Ac tracks is shown only for CD8+ memory T cells. To obtain a complete picture, the authors should also display the tracks of ATAC, H3K4me1 and H3K27Ac in naïve and memory CD4+ T cells and these histone marks in naïve CD8+ T cells. Furthermore, the authors state “the analysis of ATAC-seq database showed that the Ccl5-RBR becomes more accessible in effector/memory T cells than in naïve T cells”, but the actual difference does not look impressive. The authors should give an estimate of the extent to which it becomes “more accessible”.

We thank the reviewer for this suggestion and we agree that examining Ccl5 locus by comparing CD4$^+$ and CD8$^+$ T cells gives a more complete picture. Hence, we modified the figure to show ATAC-seq data of naïve and effector subsets of both CD4$^+$ and CD8$^+$ T cells in Fig 2A. During this analysis, we noticed that the differences in open chromatin structure are more impressive between CD4$^+$ and CD8$^+$ T cells than between effector/memory CD8+ T and naïve CD8$^+$ T cells as the reviewer suggested. We therefore changed the text and figure accordingly (page 6, lines 132-134).

2. Page 6 line 128: The Ccl5-RBR actually contains four RRS, not just the two marked in Fig. S3B and S3C and schematically illustrated in Fig. 3A. An additional ACCACA is found 50 bp downstream of the first ACCACA that is marked in bold letters and another RRS, TGCGGT, is adjacent to the second marked ACCACA.

We thank the reviewer for pointing out other possible RRSs in the Ccl5-RBR. Among several different criteria to formulate RUNX binding motifs, we used 7 nt consensus motif of 5'-PuACCPuCA-3', not 6 nt motif of 5'-ACCACA-3' that yields 2 additional RUNX binding motifs. If the reviewer insists that ACCACA is more appropriate, it is fine for us to include other two RSSs in the manuscript.

3. Page 6 line 129: The authors mention rs11652536, a SNP located in CCL5-RBR, that is associated with the severity of liver fibrosis in humans. The relevance of this statement to the data in this manuscript is unclear and could be misleading, since it is completely unknown whether this SNP affects CCL5 expression in human T or NK cells that are the subject of the present work and whether the liver fibrosis is mediated by these immune cells. Also, this SNP was not reported in a genome-wide associated study.

We thank the reviewer for this thoughtful suggestion. We agree with the review's comment that mentioning of this SNP would be misleading. We therefore eliminated the description of the SNP from the manuscript.

4. Page 6 line 140: In Fig. 2C, the heading should be changed from "Activated T cells" to "Activated CD8 T cells".

We thank the reviewer for the suggestion. We now modified the text accordingly (page 7, line 165).

5. Page 6 line 153: The section is written in a convoluted way. To clarify the authors should instead of "Spleen" in Figures 2B and 3B, show data in CD4+ and CD8+ T cells, as they did in Figure 1.

We thank the reviewer for the suggestion. We now modified the figures and the text accordingly.

6. Page 6, line 162: It is unclear whether the cells referred to as "CD44+ memory-like T cells" include CD4+CD44+, CD8+CD44+ or both cell types. This point is important as expression of Ccl5 in activated CD4+ T cells differs considerably from that in activated CD8+ T cells (Fig. 3D). Presumably, the spleen cells depicted as "TCR [illegible] of+" in the 2 right pa[illegible] Fig. 3B are CD8+CD44+, if so, they should be mark "TCR [illegible] +CD8+. The[illegible] in all places where CD44+ cells are mentioned, whether they refer to CD4, CD8 or to both types of memory-like T cells.

We really appreciate the reviewer for the careful reading of our manuscript. We now corrected the description of Figure 3B as $CD8^{+}$. And we removed unnecessary uses of "CD44 expression" in this paragraph.

7. Page 8 line 208: It is unclear from the 3C assay how Ccl5 expression was increased in CbfF/F;Cd4-Cre in activated CD4+ T cells (Fig. 3D), while there were merely a minor signal in the 3C assay between the Ccl5 promoter and the Ccl5-DE in these cells (Fig. 4C). The authors should clarify this ambiguity.

We thank the reviewer for this thoughtful comment. Our genetic results show that Ccl5 expression is induced from activated $CD4^{+}$ T cells when Cbfb was deleted. This Ccl5 expression was still observed even in the absence of Ccl5-PE ($Ccl5^{\Delta PE/\Delta PE}$;$Cbfb^{F/F}$:Cd4-Cre mice). To interpret this finding, we hypothesized that other putative enhancers responsible for Ccl5 expression in activated T cells are also repressed by Runx/Cbfb, letting us to combine enChIP-seq data with Runx/Cbfb-ChIP seq data to identify the Ccl5-DE. Indeed, Ccl5-DE consists of two elements, DEa and DEb, the latter has the repressive activity, contains RSSs and is bound by Runx/Cbfb. Having these results, we think that one possible mechanism would be that Runx/Cbfb represses the Ccl5-DE activity in activated $CD4^{+}$ T cells. Our new 3C results also indicate that Runx/Cbfb is not required for a long-chromatin looping between Ccl5 promoter and the Ccl5-DE in activated $CD8^{+}$ T cells. Unfortunately, our 3C assay is not sensitive enough to detect this looping in activated $CD4^{+}$T cell population in which CCL5 is expressed only in 2% cells. We think a minor signal in the 3C assay would not formally exclude our explanation.

8. Page 9 line 218-219: In activated CD8+ T cells; DEb element has a clear repressive activity on Ccl5 expression as depicted in Fig. 4D. But besides showing CBFb binding to this element (ChIP-seq Fig. 4A), no data is given as to the presence of RRS in this region, nor for the effect of introducing a DEb-specific mutation in such an RRS. It is thus unclear whether DEb repressive activity depends on CBFb binding to this element. Consequently, the conclusions in the Abstract (lines 22-23) and Introduction (page 4 lines 69-70) that "both enhancers are negatively regulated by RUNX/CBFb complexes" have not been established experimentally and the DEb element function is not characterized. These flaws should be amended.

We really appreciate the reviewer for the thoughtful suggestion. Now we show the sequences of the DEb element in Fig. S4 and marked the positions of two RRSs in DEb element in Fig. S4 C with bold font. We agree that generating more gene modified mouse strains could be important to definitely prove the function of these RSSs in regulating DEb silencer activity as well as in recruitment of RUNX/CBFb. However, generation and characterizations of mouse strains with those mutations will require at least one more year. We hope that the reviewer agree that this experiment is beyond the scope of the current study and our current genetic and ChIP results are supportive enough to propose both enhancers are negatively regulated by RUNX/CBFb complexes.

9. Page 9 lines 222-238: The authors conclude that the looping of the Ccl5 promoter with Ccl5-DE is dependent on Satb1 which presumably associates with Cbfb at the Ccl5-DE. They show that this Satb1-mediated looping is responsible for the enhanced expression of Ccl5 in Satb1F/F;Cd4-Cre CD8+ T cells. What is not demonstrated and critically missing is an experiment showing that the same holds for CbfF/F;Cd4-Cre CD4+ and/or CD8+ T cells. It is particularly important because Ccl5-DE does not harbor a RRS, as stated in the Discussion. The reader would also be interested to know whether the Ccl5 promoter bears a RRS.

We thank the reviewer for this critical suggestion. Now we performed additional 3C assays using CBFb-deficient cells and added results in Fig. 4 C in the revised manuscript. Our new results indicate that loss of CBFb expression does not affect the chromatin looping. Also, the promoter area (200 bp upstream and 200bp downstream of the TSS of Ccl5) does not harbor either 5'-ACCACA-3' nor 5'-PuACCPuCA-3'. We described this in the result section (Page 6 lines 138-139).

10. Page 9 line 234 (related to Fig. 5D): In the Figure itself Ccl5 expressing cells in Satb1F/F; CD4-Cre CD44+ CD8+ T cells appears to be increased ~5-fold, yet in the text it is written "Homeostatic Ccl5 expression in CD44+ memory CD8+ T cells was unaffected, or tended to increase in Satb1-deficiency". This discrepancy should be dealt with. Also, Fig. 4D does not include a histogram to show the statistics of the data depicted as a dot plot.

We thank the reviewer for this suggestion and apologize for unclear description. As we reported previously (Cell Rep.;19:1176. 2017), SATB1 knockout mice showed an increase of $CD44^+$ $CD8^+$ T cells probably due to autoimmune phenotypes. Since CCL5 is mostly expressed by $CD44^+$ $CD8^+$ T cells (Fig. 1 B), this increase of $CD44^+$ T cells in the absence of SATB1 also resulted in an increase of the percentage of $CCL5^+$ cells in total T cell population. Therefore, we thought that it is fair to compare CCL5 expression in $CD44^+$ T cell population and examined the ratio of $CCL5^+CD44^+$ cells to $CCL5^-CD44^+$ cells. We found that this ratio tended to increase in SATB1-deficienct cells with 0.17 to 0.28. We modified the text and the figure to clarify this point (Page 10 lines 251-253).

11. Page 10 lines 263-265: The finding that in lung NK "about two thirds of total mRNAs either increased or decreased by the absence of Ccl5-PE" is quite surprising and very unusual. The authors should analyze whether this applies also to mRNAs of the three Runx genes Runx1, Runx2 and Runx3 and whether they are increased or decreased? It is also, important to demonstrate haw expression of these genes is affected in Ccl5-PE-deficient activated CD4+ and CD8+ T cells? This is particularly important for Runx3 known to regulates proliferation of activated NK, CD4+ Th1 and CD8+ T cells.

We thank the reviewer for this suggestion. We were also surprised to find such large differences in mRNA levels in the absence of Ccl5-PE. As the reviewer asked, we looked into mRNA of RUNX and CBFb genes, and found that there are no significant changes in their expression in lung NK cells in the absence or presence of Ccl5-PE (below figure). Therefore, we believe that it is unlikely that the properties of lung NK are changed through altering levels of RUNX expression. As a side note, we also confirmed that RUNX levels are not significantly changed in Ccl5-PE-deficient activated $CD4^+$ and $CD8^+$ T cells (below figure).

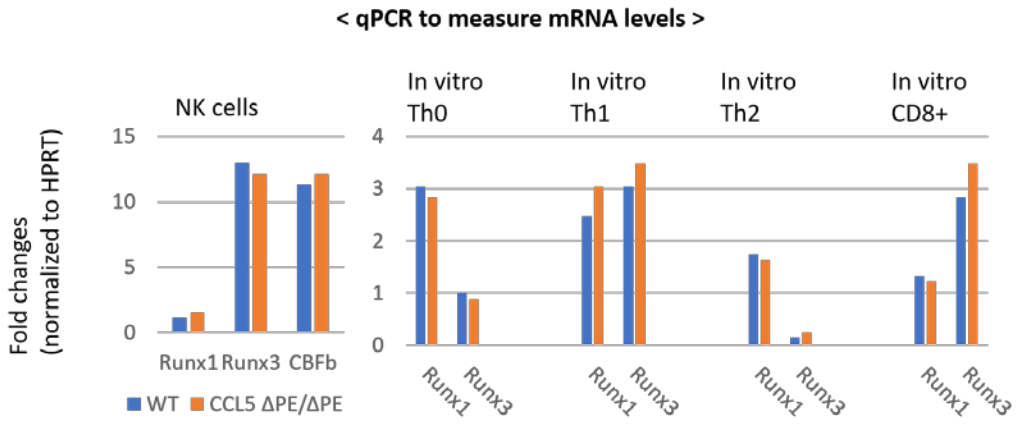

12. Page 12 lines 312-314: The authors should demonstrate whether the conclusion about the impact of blocking Ccl5 expression in lung NK-mediated killing of melanoma cells also apply to CD8+ T cells.

We thank the reviewer for the suggestion. However, since the B16 cancer model we used lacks MHC class expression, we think that it is unlikely that CD8^{+} T cell-mediated killing is involved in rejection of B16 melanoma.

13. Page 12 lines 321-329: It is entirely unclear how the authors envision the mechanism undelaying enhancers-mediated regulation of Ccl5 expression by Satb1 and Cbfb in a situation where the Ccl5-DE lacks an RRS, but Cbfb is required for repressing Ccl5 expression in NK and T cells.

In this context, the statement in lines 327-328 “The lack of RUNX-recognition sites in the Ccl5-DE indicates that the ways by which RUNX-CBFb complexes are recruited to the two Ccl5 enhancers presumably differ” is not sustained and thus vague.

We thank the reviewer for the thoughtful insight which definitely advances our discussion on the mechanism of CCL5 regulation. Even though the enhancer Ccl5-DEa lacks Runx Recognition Sites (RSS), the adjacent Ccl5-DEb with silencer function contains two RSSs. We previously reported that ThPOK, another target gene of Runx, also has a Runx-depedent silencer nearby an enhancer (J Immunol. 2013 Feb 15;190(4):1397-401). Therefore, we propose that the distal enhancer Ccl5-DEa is indirectly suppressed by the nearby silencer Ccl5-DEb, while the proximal enhancer Ccl5-PE could be antagonized by RUNX/CBFb via RSSs within the Ccl5-PE, In order to clarify this point, we revised the text (page 14, lines 358-361).

14. Finally, while the title of the manuscript implies an important role for RUNX in suppressing Ccl5 expression, in the discussion (page 13 line 330) it is stated “It is unclear how RUNX-mediated suppression is cancelled in Ccl5 expressing cells”. The authors should consider rephrasing their conclusions and title to reflect the finding described in the manuscript.

We thank the reviewer for the thoughtful suggestion. However, we are afraid that the reviewer might have been misled. It is clear that RUNX represses Ccl5 expression and our study has provided an important piece to the regulatory network governing Ccl5 expression by showing evidences for the negative regulator of Ccl5. On the other hand, we also discussed how RUNX-mediated repressive mechanism is antagonized to activate Ccl5 gene. This is a mirror image to consider how Ccl5 expression is induced by antagonizing negative regulation. We believe that it is worth discussing this point. However, we agree that our previous way to discuss this point was unclear and revised the text (page 14, lines 358-361).If the reviewer considers that this discussion is still confusing to the readers, we are willing to eliminate it.

Minor:

1. Page 4 line 79: Add the word “factor” after “transcription”.

2. Page 5 line 109: Remove the word “of” before “in suppressing CC chemokines”.

3. Page 6, line 155: the word “CD8” after “activated” should be added.

4. Fig. 3B right panel histogram: The red triangles (CbfbF/F; CD4-Cre) and the blue squares (Ccl5 □PE/□PE) should be switched.

We thank the reviewer for the careful reading of our manuscript. We changed the text and figures accordingly.

Reviewer #2 (Cancer immunity, NK, transcription regulation) (Remarks to the Author):

The authors investigate the regulation of CCL5 by naïve (homeostatic expression) vs activated (induced expression) T cells and the role of Runx proteins in this context. They also study how homeostatic CCL5 expression impact on anti-tumor immunity in the context of B16 melanoma.

The authors claim the identification of two enhancers: one that is involved in the regulation of the homeostatic expression of CCL5 and termed CCL5-RBR or CCL5-PE, and one that is involved in the regulation of the induced expression of CCL5, termed CCL5-DE.

The MS provides an interesting hypothesis, but the data provided need to be substantiated and clarified to better support it.

We wish to express our appreciation to the reviewer for their insightful comments on our paper. The comments have helped us significantly improve the paper.

General comments

In this work, the authors produce a large series of 6 novel mouse mutants, including BAC transgenic mutants and gene targeted mice using the Crispr/Cas9 technology. This a huge effort. However, the authors do not provide any characterization of the impact of the gene modifications on the immune system in those mice so that the usefulness of the gene targeted mice is limited (more details below). The accessibility of data would gain substantially, if the authors would unify the presentation of the data. In figure 1 the expression of CCL5 is shown for CD4+CD8-TCRb+ and CD8+TCRb+ T cells (Figure 1b middle histograms). The analysis of the latter cells is more detailed: expression of CCL5 in relation to expression of CD44 (Figure 1b, right dot plots). In subsequent figures, the representation changed as CCL5 or CCL5-GFP expression by

all TCRb+ cells are shown in relation to CD44: Figure 2B and C, Figure 3 B, Figure 5D, FS1C. It is not explained why this is so. The authors need to state if the observed effects are similar (or not!) in CD4 as well as CD8 T cells and provide those data on CD4 and CD8 T cells, separately, in a supplemental figure. Moreover, when the authors analyze activated T cells, they separate CD4 from CD8 T cells.!

We thank the reviewer for the suggestion that is similar to the reviewer #1. We showed only $CD8^+$ T cells in some cases due to two reasons. One is because most of CCL5 expression in ex vivo cells is from $CD8^+$ T cells. Another reason is that CBFb and SATB1 knockout mice do not have the normal separation of CD4 and CD8 expression, so that it is difficult to clearly divide T cells into traditional MHCII-restricted CD4+ helper and MHCI-restricted CD8+ cytotoxic T cells. Nonetheless, according to the reviewers' suggestion, we reanalyzed/reorganized figures. Now we show T cells subsets by $CD4^+$ and $CD8^+$ instead of $TCRb^+$ in Fig. 2B, 2C, 2D, 3B for smoother presentation of our data.

It is not always clearly stated how many independent experiments have been performed. Please, rectify, where the information is missing.

In order to show the numbers of independent experiments or the numbers of mice analyzed, we included the summary graphs in which individual dot represents independent experiment or mouse analyzed. We added a sentence also in the method section to indicate this.

Specific comments/The data will be reviewed figure by figure:

Figure 2

Why do the authors use different mouse mutants when analyzing the homeostatic expression of CCL5 (CCL5-GFP-ΔRBR) vs induced CCL5 expression (CCL5-GFPRBRM)?

CCL5-GFP-ΔRBR mice have been deleted for the whole RBR region encompassing the proximal enhancer and were used to analyze the function of the total RBR region. On the other hand, CCL5-GFP-RBR^M mice have specific point mutations on the Runx Recognition Sites (RRS) within the RBR to examine function of RRSs. By using these two independent strains, we found that RRSs function as a negative regulator for enhancer function of the RBR. In order to make this point clearer in the figure as the reviewer suggested, we eliminated the plot showing CCL5 expression from activated T cells of CCL5-GFP-ΔRBR mice and modified the text accordingly.

In naïve mice, CCL5-GFP expression is abolished in absence of the identified enhancer (CCL5-GFP-ΔRBR), but how is it when the enhancer is present, but only the two binding sites for the Runx repressor (RRS) are mutated? One would expect an increased CCL5-GFP expression - actually, as seen in the activated T cells from these mice (CCL5-GFPRBRM). The analysis of naïve CD8 T cells from CCL5-GFPRBRM would also help interpret the data derived from activated T cells.

According to the reviewer's suggestion, we modified the Fig. 2 and now show CCL5-GFP expression in naïve $CD8^+$ T cells from CCL5-GFP-RBR^M mice (Fig. 2C). As can be seen from the revised figure, CCL5-GFP-RBR^M mice show an increase of CCL5-GFP during the steady state.

How do the authors explain that CCL5-GFP is not expressed in activated T cells (- absence of CCL5-DE), while in absence of RUNX function (Cbfb mutation) it is induced – in an apparent absence of other Runx-ChIP peaks with RRS and open in effector/memory cells?

We thank the reviewer for asking this interesting question. Our results shown in Fig. 2D indicate that the CCL5-GFP expression is also observed from CCL5-GFP-RBR^M mice, as was observed from the CCL5-GFP BAC Tg upon the loss of Cbfb expression. Taken these results together, one possible mechanism would be that RUNX binding to the CCL5-PE might regulate not only the cell-type specificity but also the functional stage-specificity of the CCL5-PE. In other words, RUNX association with the CCL5-PE could represse the enhancer function also in activated $CD8^+$ T cells.

Figure 3

Generation of CCL5ΔPE/ΔPE mice = mice carrying a deletion that removes both RSS sites from the RBR region in the identified CCL5 enhancer. CCL5ΔPE/ΔPE mice show a similar phenotype than CCL5-GFP-ΔRBR (and naïve CCL5-GFPRBRM also?) mice. Again, how do the authors explain that CCL5 is expressed in naïve T cells from CCL5ΔPE/ΔPE mice when Cbfb is deleted – i.e., the repressor and its binding site are missing? The last phrase on page 7 (line 173 onwards) is not clear.

We thank the reviewer for asking this question and are sorry for insufficient description on page 7. We observed CCL5 expression from activated $CCL5^{\Delta PE/\Delta PE}$ cells upon the loss of Cbfb. This finding suggests that the CCL5 expression, which is controlled independently of the Ccl5-PE and presumably by the putative Ccl5-DE, is also repressed by Runx/Cbfb complexes. Our characterization of the Ccl5-DE region indeed found that the nearby DEb element has a

suppressive function against the enhancer function of the DEa element (Fig 4D, E). Therefore, one possible explanation is that the loss of RUNX/CBFb-mediated repressive mechanism on the Ccl5-DEb induces Ccl5 expression from CCL5$^{\Delta PE/\Delta PE}$ cells, although there remain possibilities that other uncharacterized Runx Binding Regions responsible for such CCL5 expression. We modified the phrase to increase clarity (page 8,line 186-187).

B: In the diagram on the right, the red triangles and blue squares are mixed up.

Also, please, unify the color code in this figure aside from the mix-up. This would enhance readability of the data.

We thank the reviewer for the careful reading our manuscript. We corrected the error.

C: On the right “total” splenocytes are shown where it should be TCRb- splenocytes in order to restrict the analysis on NK cells and to remove NK-T cells.

We thank the reviewer for the suggestion. The aim of this figure is to examine Ccl5 expression in gdT cells and NK cells. In order to specify NK cells more specifically, we reanalyzed with TCRb-negative gate and confirmed that NKT cells do not express CCL5 during the steady-state and Ccl5 expression is mostly restricted to NK cells within NK1.1^{+} population. We edited the text to indicate this.

The legend of the y-axes on both diagrams needs to be corrected and completed.

We thank you the reviewer for the careful reading our manuscript. We corrected the error.

Figure 5

C: A negative control is missing to be able to interpret the data shown: do all CD8+ T cells from Sat1b1f/f;Cd4-cre mutant mice express IFNgamma upon stimulation but at a lower level than WT T cells or do only 60% of the activated mutant CD8+T cells express it?

In our hands, most (80-90%) of wild-type CD8^{+} T cells express IFNγ after 5-6 days of stimulation. On the other hand, IFNγ expression is limited to 60 % of SATB1-deficient activated CD8^{+} T cells. However, the ratio of CCL5^{+} cells to CCL5^{-} cells in IFNγ^{+} population is still

significantly reduced in Satb1-deficient CD8^{+} T cells, which is supportive to our interpretation. Also, we added a panel showing isotype control of IFNg staining as the reviewer asked in Fig. 5 C.

D: CD8+ T cells from Sat1b1f/f;Cd4-cre mutant mice seem to harbor an increased CD44+ population. Are these cells pre-activated? Please, provide data on their activation status compared to WT cells. Please, provide statistics of the % of CCL5+ cells among naïve CD44+CD8+ T cells. What means “multiple independent experiments” in the figure legend? Give numbers.

We apologize for the confusion. This point is related to the point #10 raised by the reviewer #1. As we replied to the reviewer #1, SATB1 knockout mice showed an increase of CD44^{+} CD8^{+} T cells probably due to autoimmune phenotypes (Cell Rep.;19:1176. 2017). Since CCL5 is mostly expressed by CD44^{+} T cells (Fig. 1 B), this increase of CD44^{+} CD8^{+} T also resulted in an increase of the percentage of CCL5^{+} cells in total T cell population. Therefore, we thought that it is fair to compare CCL5 expression in CD44^{+} T cell population and examined the ratio of CCL5^{+}CD44^{+} cells to CCL5^{-}CD44^{+} cells. Now we added the summary graph showing n values of the experiment in Fig. 5 D.

Figure 6

A: Surprisingly, in the controls Trm T cells seem to lack CD69 and CD103 expression. However, the majority of Trm T cells in the lung express these markers (review Mueller and Mackay, 2016). Please, comment.

We thank the reviewer for raising this question. However, as far as we know from literatures and our experiences, lungs of C57/Bl6 mice kept in clean SPF conditions have just few percentages of CD69^{+} and/or CD103^{+} Trm T cells. This is quite different from antigen-challenged (or non-SPF condition) mice that were discussed in the review by Mueller and Mackay and is also different from human lungs.

Also, the gates in the dot plot on the right are wrongly placed: they cut the populations in half. This needs to be rectified. Adjust the statistics accordingly

We thank the reviewer for the notification. We amended the error.

D: The aim is to assess the functional status of the NK cells from a novel mutant mouse line. It is surprising that the authors do not characterize the NK cells in these mice, first: their differentiation status and repertoire expression, developmental stages in the bone marrow and tr-NK cells in the liver. This needs to be done.

We thank the reviewer for this thoughtful suggestion. We examined differentiation status of lung NK cells by using CD11b and CD27 markers and now show new results in Fig. 6 C. Also the activation status assessed by IFNγ and Perforin expression is shown in Fig 6 F. As evident from these results, NK cells of those mice appear to be similar in the differentiation status during the steady state, but their functional properties become different after stimulation.

RNA-seq-based analyses allow to reveal functional potential of NK cells. The data obtained are interesting, but only suggestive as RNA expression does not necessary translates into changes on the protein level or functional consequences. In order to assess the true functional status of mutant vs control NK cells it is necessary to perform in vitro assays with purified NK cells.

We thank the reviewer for the insightful suggestion. Now we added the data of in vitro stimulation assay in which we stimulate NK cells by B16-F10 cancer cells (Fig. 6 F). Consistent with the RNA-seq results of NK cells and with results of our in vivo cancer model, NK cells of CCL5-PE knockout become more cytotoxic by producing higher IFNg and Perforin compared to wild type.

E: Why do the authors say that the difference of NK cells between WT and CCL5-PE KO mice is as much distinct as the NK cells between spleen and lung (page 11, lines 268/269)? First, the data do not support that, and second, why would that be of any interest?

We thank the reviewer for pointing out this unclear statement. Based on our PCA, lung NK cells are more separated between wild-type mice and CCL5-PE KO mice than spleen NK cells (distance of Dim2), so that what we would like to point out was that the distance between WT and KO is more pronounced in the lung than in the spleen. We corrected the text in the revised manuscript (page11, lines 284-285) .

The phrase “These findings…potent activities of NK cells” (lines 271-274) is wrong. It is neither clear at this point, if there are effectively differences, nor if they would be NK cell intrinsic or due to the aberrant activities of accessory cells.

We thank the reviewer for raising this criticism. However, we examined NK cell activity in vitro in Fig 6 F and Fig S5 C, and confirmed the alteration of NK cell function in the absence of CCL5. Thus, we believe that our statement that loss of CCL5 has significant impacts on NK cell properties is not wrong. On the other hands, we agree that our data cannot exclude a possibility that changes of NK cell properties occurred in a cell-extrinsic manner in vivo as the reviewer pointed out. Therefore, we revised the text and removed "NK cells of non-immune tissues" from previous description of "loss of CCL5 in NK cells of non-immune tissues" (Page 11, lines 287-289).

Figure 7

Line283: "Given altered properties…" this phrase is wrong! This has not been shown, only suggestive data presented (see comments Figure 6E). Add the acquired data or modify the phrase.

As we replied to the point in Fig. 6D and 6E, now we added new data of in vitro assay of NK cells in Fig. 6 F. We hope that the reviewer agrees that this new result allows us to use "Given altered properties".

The lack of a characterization of the three novel mutant mouse lines (CCL5ΔPE/ΔPE, CCL5ΔDE/ΔDE, Runx3ΔY/ΔY) does not allow to asses fully the role of CCL5 in these assays. Are only NK cells affected or also T cells (esp CD8+ T cells) or myeloid cells? Are those cells spontaneously activated? Secreting cytokines?

We thank the reviewer for this thoughtful suggestion and agree that more detailed characterizations of multiple cell types in mutant mouse lines could be more helpful to fully understand the roles of CCL5 enhancers in tumor immunity against B16-F10 melanoma models. However, the main findings of this study are identification of enhancers of the Ccl5 gene and suppressive roles of Runx proteins in Ccl5 expression. In addition, lung metastasis model using B16 melanoma which lack MHC class expression is a well-established model, in which NK cells are known to play the major role for tumor cell rejection. Therefore, we first focused on NK cells. We hope that the reviewer agrees that detailed analyses of another hematopoietic cells in these three mouse strains are out of the scope of this study and would be examined in the future study.

It seems clear that CCL5ΔPE/ΔPE mice develop fewer and Runx3ΔY/ΔY mice more B16 lung metastases than controls, but it remains unclear why. Both molecules exert activities on several cell types. A clear support for the last statement (lines 312-314) is missing.

This criticism is similar to the above criticism. Of course, we agree that CCL5 can exert its activity on other cell types in addition to NK cells in cancer microenvironments. However, we have shown that NK cells with less CCL5 expression become more cytotoxic by in vitro assays (Fig. 6 F & Fig S5 C) as well as in vivo (Fig S5 B). And, again, NK cells are shown to be the major player for rejection of B16 melanoma which lack MHC class expression (Cancer Res. 2001 Feb 1;61(3): 1095-9). We think these are supportive to our claim that NK-mediated cancer immunity is regulated by CCL5 and plays major roles in controlling B16 melanoma. However, we now have toned down and rephrased the sentence by adding "at least" (Page 13, line 334). We hope the reviewer agrees that the revised sentence is well supported with our data.

Besides providing a general characterization of the mouse mutants, it is necessary to provide more data on TIL-NK cells (absolute numbers, phenotype) and other TIL in the skin B16 experiments (Figure S5), especially TIL-CD8+ T cells (numbers and their activation status - IFNgamma expression levels, cytotoxicity).

This study was initiated to identify the transcriptional regulatory circuit which can explain stage-specific and cell-type specific expression of CCL5, because CCL5 has been shown to be an important chemokine in various immune responses. During this study, we unexpectedly found a previously unappreciated role of CCL5 that CCL5 can influence functional properties of immune cells, thus affecting cancer immunity. We plan to perform experiments to more detailed analyses of TIL-NK cells and -T cells of these knockout in the future studies, and hope that results of these experiments will be published separately in other manuscripts. We hope that the reviewer agrees that such detailed analysis of NK cells and T cells within TILs is beyond the scope of this manuscript.

FS5C: why didn't the author use NK cells from CCL5ΔPE/ΔPEand CCL5ΔDE/ΔDE mice to assess the role of CCL5 here?

We thank the reviewer for asking this point. In this assay, we addressed a direct effect of CCL5 on cytotoxicity of NK cells. For this aim, we thought that NK cells from wild type mice are sufficient. The reason is that we were afraid that NK cells from mutant mice have different cytotoxicity from the beginning and including such mutant cells might complicate the analysis.

What was the purity of NK cells used?

They were negatively selected and their purity was more than 90%..

The diagram on the right is not clear: we are looking on the ratio of annexin V+ vs annexin V- of what cells – B16 or NK cells or both? Better calculate the % of Annexin V+ B16 cells.

We thank the reviewer for the careful reading of our manuscript. The ratio of annexin V+ vs annexin V- was B16 cells. We clarified this in revised Fig S5 C.

Minor:

Typo line 109: remove “of”

We thank the reviewer for the careful reading of our manuscript. We corrected the error in text.

Line 285: what means “physiological” in this context?

We meant “meaningful” impact on cancer immunity.

Reviewer #3 (Tumor immunity, CCL5) (Remarks to the Author):

The paper of Seo, Taniguchi and colleagues reports on the novel role of RUNX/CBFb interactions in regulating host's CCL5 production and tumor growth in lung metastasis model of B16-F10. The current manuscript contains multiple interesting elements, which could help to explain current controversies regarding the roles of CCL5 and CCR5 in cancer immunity and cancer progression. Unfortunately, in its current form, the paper does not provide a clear picture and offers only very limited connection between different reported findings. Addressing the following issues could enhance the appeal and predicted impact of the paper:

1) The narrative of the paper (including the abstract) is dense and does not present a clear story. A significant amount of editing could help to highlight the key elements of the paper and connect its different element.

We thank the reviewer for this pertinent comment. We reorganized and amended the abstract for clearer delivery of our message.

2) The key proposal (title) that the modulation of CCL5 by RUNX/CBFb is responsible for the observed differences in B16-F10 growth is not fully supported by data, and seems to be based on the observed correlations rather than mechanistic evidence. Experiments showing that the RUNX status regulates the B16 growth rate only in the absence but not in the presence of CCL5 (or CCR5) blockade, would help to support the central message. Development of double knockout mice deficient in both RUNX and CCL5 could provide even stronger evidence.

We thank the reviewer for the critical suggestion. The similar concern was also raised by other reviewers. Therefore, we now show that NK cells without CCL5 are indeed more cytotoxic when cultured with cancer cells in vitro to provide a better mechanistic evidence in new Fig 6 F. We believe that this new data along with another in vitro assay in Fig S5 C together support our

claim that NK cells in the absence of CCL5 are more cytotoxic. We also provided data showing an essential requirement for RUNX in repressing Ccl5 levels and provide mechanistic insights into how Runx achieves Ccl5 repression via antagonizing two enhancers. To convey these findings to readers, we chose our current title. However, we are willing to further discuss for a better title.

We also agree with the reviewer that generating double knockout mice deficient for both RUNX and CCL5 are more supportive of our data and to clarify how much Ccl5 dysregulation is involved in reduced tumor immunity in the RUNX3 mutant.. However, generation and characterization of double mutant mice by mouse crossing will take at least a year. We hope the reviewer agrees that our current data bring significant advances to our understanding of Ccl5 gene regulation, which is worth to be published in a timely manner.

3) Taking into account that the paper addresses the role of host-derived CCL5, it is unclear why only a single model (i.v. B16-F10) is used to support the central message of the paper and evaluate the impact on tumor growth.

We thank the reviewer for the critical suggestion and strongly agree with that testing other cancer models is very important. Therefore, now we performed the requested experiment by using another well-recognized cancer model with YAC-1 cells and show the results in Fig. 7 D. This new result also showed enhanced tumor immunity in CCL5-PE KO mice as were in B16-F10 model, thus strenghthning our claim.

4) The paper implicates that the impact of RUNX is mediated by altered immune status is based on the observed associations, but lacks comparison of immunodeficient and immunocompetent animals.

We thank the reviewer for this suggestion, but we are afraid that the reviewer might have been misled. We showed altered immune status of host cells by the lack of Ccl5-enhancers. We did not show much results by using Runx-mutant mice. Also, we now show that NK cell development is not altered in Ccl5-enhancer KO mice in new Fig 6 C. We are puzzled by the reviewer's comment on this point about what kinds of immunodeficient and immunocompetent animals should be compared.. We are willing to compare our models to those animal models if this comparison is necessary to support our study.

5) The introduction of the paper very dense and describes multiple, apparently contrasting earlier reports on the role of CCL5 in antitumor immunity, but, in its current form, it does not provide the reader with a hypothesis leading the authors to the proposed studies. Similarly, the discussion

could also be enhanced to provide the reader with better understanding of how the current findings help to interpret the previously reported data.

We thank the reviewer for positive evaluation of our dense data set and for the suggestion. We amended the introduction and discussion parts for clearer delivery of our message. For instance, we added sentences to better contrast our study with previous studies (page 15, line 404-408).

6) I suggest to discuss the current observations in the context of the seminar report from Jurgen Becker showing that the presence of the CCR5Delta32 polymorphism in patients with stage IV melanoma results in a selectively decreased survival following immunotherapy, although it does not affect the melanoma survival in untreated patients (Cancer Immunol Immunother. 2008 May;57(5):685-91.)

We thank for the reviewer for such a productive suggestion. We were aware of the literature, but unable to incorporate it due to the space limitation. Now we added the discussion of the suggested literature (page 16, line 429-433).

REVIEWERS' COMMENTS:

Reviewer #2 (Remarks to the Author):

The authors have sufficiently addressed the questions raised. The modifications made to the manuscript, text as well as the figures, well improved the work.

Reviewer #3 (Remarks to the Author):

The revised paper of Seo, Taniguchi and colleagues reports on the novel role of RUNX/CBFb interactions in regulating host's CCL5 production and tumor growth in lung metastasis model of B16-F10. The revised manuscript contains multiple interesting elements, and addressed some of my concerns, but still does not deal with several of the key issues which undermine the central message:
1) The central proposal (title) that the immune modulation of CCL5 by RUNX/CBFb is responsible for the observed differences in B16-F10 growth in vivo is still not supported by data, and remains to be based on the observed correlations rather than mechanistic evidence. Experiments showing that the RUNX status regulates the B16 growth rate only in the absence but not in the presence of CCL5 (or CCR5) blockade, would help to support the central message. Development of double knockout mice deficient in both RUNX and CCL5 could provide even stronger evidence. The authors kindly acknowledge my original request for such data in my original review but, simply provide more in vitro data on NK cell function and argue that providing definitive in vivo data liking immune phenomena to cancer progression would require too much of time. I remain concerned that in the absence of these data the central message of the paper lacks key experimental support. It is still unclear if the changed status of any of the immune cells studied in the paper is indeed responsible for the changes in the kinetics of tumor growth.
2) In my original review, I suggested to discuss the current observations in context of the apparently contradictory human data from patients with CCR5Delta32 mutation which have decreased survival following immunotherapy (Cancer Immunol Immunother. 2008 May;57(5):685-91). Again, the reviewers acknowledge this request but argue against discussing that paper citing lack of space.

REVIEWERS' COMMENTS:

Reviewer #2 (Remarks to the Author):

The authors have sufficiently addressed the questions raised. The modifications made to the manuscript, text as well as the figures, well improved the work.
We thank the reviewer.

Reviewer #3 (Remarks to the Author):

The revised paper of Seo, Taniguchi and colleagues reports on the novel role of RUNX/CBFb interactions in regulating host's CCL5 production and tumor growth in lung metastasis model of B16-F10. The revised manuscript contains multiple interesting elements, and addressed some of my concerns, but still does not deal with several of the key issues which undermine the central message:
1) The central proposal (title) that the immune modulation of CCL5 by RUNX/CBFb is responsible for the observed differences in B16-F10 growth in vivo is still not supported by data, and remains to be based on the observed correlations rather than mechanistic evidence. Experiments showing that the RUNX status regulates the B16 growth rate only in the absence but not in the presence of CCL5 (or CCR5) blockade, would help to support the central message. Development of double knockout mice deficient in both RUNX and CCL5 could provide even stronger evidence. The authors kindly acknowledge my original request for such data in my original review but, simply provide more in vitro data on NK cell function and argue that providing definitive in vivo data liking immune phenomena to cancer progression would require too much of time. I remain concerned that in the absence of these data the central message of the paper lacks key experimental support. It is still unclear if the changed status of any of the immune cells studied in the paper is indeed responsible for the changes in the kinetics of tumor growth.

We really appreciated the reviewers' suggestion that the generation of the double knockout for RUNX and CCL5 can be more useful. However, clearly this experiment will take more than another year. Alternative approach to confirm the involvement of CCL5-CCR5 axis in enhanced metastasis of B16-F10 melanoma in Runx mutant mice could be using reagents that block CCL5-CCR5 axis, such as anti-CCL5 Ab or CCR5 inhibitor Maraviroc. However the systemic administration of these reagents has a problematic drawback due to multiple functions of CCL5-CCR5 axis in anti-tumor responses. CCL5 from immune cells has been known as an anti-cancer cytokine. On the other hand, recent studies have proposed a new role of host-derived CCL5 that supports survival of cancer cells. Thus, blocking reagents of CCL5-CCR5 axis work on both immune cells and cancer cells, making the interpretation of results very complicated. Therefore, we hesitated to use such reagents.

By using Ccl5 enhancer knock-out mice, our results shown in this manuscript bring strong evidence to verify the role of host CCL5 as a pro-cancer molecule.

Therefore, we believe that our results clearly support our claim that tumor immunity is enhanced by reduced Ccl5 expression in host immune cells. The other important finding in our study is the identification of repressive mechanisms for Ccl5 expression, which are medicated by Runx proteins. To convey these two messages in the title, we carefully consider the final title after taking account for the editor's suggestion.

According to the reviewer's criticism and editor's suggestion to tone down about the role of CCL5 and Runx during the tumorigenesis, we also modified the Abstract. Again, the main message of the manuscript is the discovery of the regulatory mechanism of CCL5 by the two enhancers with an unique stage-specificity and negative regulatory roles of RUNX on these enhancers. We hope the reviewer understand our intention and agree with the revised Title and Abstract.

2) In my original review, I suggested to discuss the current observations in context of the apparently contradictory human data from patients with CCR5Delta32 mutation which have decreased survival following immunotherapy (Cancer Immunol Immunother. 2008 May;57(5):685-91). Again, the reviewers acknowledge this request but argue against discussing that paper citing lack of space.

We afraid that the reviewer might have missed the newly added sentences describing CCR5Delta32 in the discussion section. We now further discuss the CCR5Delta32 mutation in the discussion section. Thank you.
